# Revised Phylogeny and Novel Horizontally Acquired Virulence Determinants of the Model Soft Rot Phytopathogen *Pectobacterium wasabiae* SCC3193

**DOI:** 10.1371/journal.ppat.1003013

**Published:** 2012-11-01

**Authors:** Johanna Nykyri, Outi Niemi, Patrik Koskinen, Jussi Nokso-Koivisto, Miia Pasanen, Martin Broberg, Ilja Plyusnin, Petri Törönen, Liisa Holm, Minna Pirhonen, E. Tapio Palva

**Affiliations:** 1 Department of Agricultural Sciences, Plant Pathology, University of Helsinki, Helsinki, Finland; 2 Department of Biosciences, Division of Genetics, University of Helsinki, Helsinki, Finland; 3 Institute of Biotechnology, University of Helsinki, Helsinki, Finland; Oregon State University, United States of America

## Abstract

Soft rot disease is economically one of the most devastating bacterial diseases affecting plants worldwide. In this study, we present novel insights into the phylogeny and virulence of the soft rot model *Pectobacterium* sp. SCC3193, which was isolated from a diseased potato stem in Finland in the early 1980s. Genomic approaches, including proteome and genome comparisons of all sequenced soft rot bacteria, revealed that SCC3193, previously included in the species *Pectobacterium carotovorum*, can now be more accurately classified as *Pectobacterium wasabiae*. Together with the recently revised phylogeny of a few *P. carotovorum* strains and an increasing number of studies on *P. wasabiae*, our work indicates that *P. wasabiae* has been unnoticed but present in potato fields worldwide. A combination of genomic approaches and in planta experiments identified features that separate SCC3193 and other *P. wasabiae* strains from the rest of soft rot bacteria, such as the absence of a type III secretion system that contributes to virulence of other soft rot species. Experimentally established virulence determinants include the putative transcriptional regulator SirB, two partially redundant type VI secretion systems and two horizontally acquired clusters (Vic1 and Vic2), which contain predicted virulence genes. Genome comparison also revealed other interesting traits that may be related to life in planta or other specific environmental conditions. These traits include a predicted benzoic acid/salicylic acid carboxyl methyltransferase of eukaryotic origin. The novelties found in this work indicate that soft rot bacteria have a reservoir of unknown traits that may be utilized in the poorly understood latent stage in planta. The genomic approaches and the comparison of the model strain SCC3193 to other sequenced *Pectobacterium* strains, including the type strain of *P. wasabiae*, provides a solid basis for further investigation of the virulence, distribution and phylogeny of soft rot bacteria and, potentially, other bacteria as well.

## Author Summary

The soft rot bacteria of the genus *Pectobacterium* are an economically important group of plant pathogens in the Enterobacteriaceae family. Pectobacteria are characterized by their massive production of plant cell wall-degrading enzymes, which are aggressively used to decay the living plant tissue for nutrient acquisition. Genome analysis of *Pectobacterium* sp. SCC3193, a genetically well characterized model in soft rot research, provided several surprises. Phylogenetic analysis demonstrated that SCC3193 belongs to the species *Pectobacterium wasabiae*, which has recently been reported from potato in increasing numbers worldwide. It is not clear whether *P. wasabiae* represents a true emerging pathogen in potato fields or whether traditional methods have been unable to differentiate between *P. wasabiae* and *P. carotovorum*. The genome of SCC3193 was found to harbor novel virulence determinants, some of which may have been acquired from distantly related bacterial species or even from other kingdoms, such as plants. The 25 years of SCC3193 research and the novelties found in this study will provide the basis for understanding the host pathogen interactions of *P. wasabiae* and improving strategies to combat bacterial soft rot.

## Introduction

The soft rot bacteria *Pectobacterium* and *Dickeya* of the family Enterobacteriaceae are responsible for significant, global economic losses of crops and ornamental plants, both in the field and in storage. Although soft rot enterobacteria can infect a wide variety of plants, the main crop affected is potato (*Solanum tuberosum* L.). Potato is number five among food crops in the world and is a staple food in a number of countries (FAOSTAT 2009, http://faostat.fao.org/). Previously, the *Pectobacterium* and *Dickeya* species were classified into the *Erwinia* genus, and relatively recent phylogenetic analyses have elevated them to novel genera [1], [2]. In addition, some of the subspecies have been raised to the species level [3]. For historical reasons, three distinct potato diseases caused by soft rot enterobacteria are described: common soft rot (tuber symptoms), blackleg (tuber-born stem disease) and aerial stem rot (spread mechanically or via insects) [4]. *Pectobacterium* and *Dickeya* are characterized as opportunistic pathogens that switch from an asymptomatic latent phase into a virulent phase in suitable environmental conditions [5]. The virulent phase is thought to begin under anoxic conditions when oxygen radical-dependent plant defense mechanisms decline, allowing bacterial multiplication and the induction of plant cell wall-degrading enzymes (PCWDEs). The induction of PCWDEs occurs when the bacterial cell density reaches a quorum of 10^7^ cfu/g of plant tissue [5].

The soft rot enterobacteria are necrotrophs and are generally considered to be brute-force pathogens relying on PCWDEs for pathogenicity. In fact, several regulatory mutants affecting enzyme production are essentially avirulent [6]–[9]. Studies of more fine-tuned virulence mechanisms may have been hampered by this massive production of PCWDEs [6]. Therefore, the latent stage preceding necrotrophy remains poorly understood. However, during the last 25 years, a number of other virulent lifestyle promoting determinants have been identified from *Pectobacterium*. These determinants include the following: flagella-based motility, cell membrane structures, such as enterobacterial common antigen (ECA) and lipopolysaccharide (LPS), type III secretion systems (T3SS), type IV secretion systems (T4SS), type VI secretion systems (T6SS), necrosis-inducing protein (Nip), a protein similar to an avirulence protein in *Xanthomonas* (Svx), coronafacic acid synthesis pathways (*cfa* genes), plant ferredoxin-like protein (FerE) and citrate uptake and 3-hydroxy-2-butanone pathways (*bud*) [10]–[19]. The virulence strategies of necrotrophic bacteria differ from hemibiotrophic and biotrophic phytopathogens, such as *Erwinia amylovora*, *Pantoea* sp. and *Agrobacterium tumefaciens*, which rely mainly on T3SS or T4SS for pathogenicity [20], [21]. The genomic era has provided novel tools for the identification of previously unknown virulence determinants without large-scale biological experiments. Several genomic studies of plant and animal pathogenic enterobacteria have been conducted, but only two have been published on soft rot bacteria. The first study compared *P. atrosepticum* and *Salmonella*, and the second study characterized *P. atrosepticum*, *P. carotovorum* and *P. carotovorum* subsp. *brasiliensis* [11], [22].

The soft rot pathogen *Pectobacterium* sp. SCC3193 was originally isolated from a diseased potato stem from a Finnish field in the early 1980s and was characterized as *P. carotovorum* (previously called *Erwinia carotovora* subsp. *carotovora*) [23]. Since its discovery, SCC3193 has been a model strain in soft rot research, and much is known about its virulence and molecular biology [7], [15], [17], [23]–[33]. In this work, we present a revised phylogeny, an analysis of virulence determinants and a characterization of genomic islands; we also discuss novel virulence strategies utilized throughout the lifestyle of *Pectobacterium* sp. SCC3193. We show that SCC3193 can be taxonomically classified as *Pectobacterium wasabiae* and that *P. wasabiae* has unique features when compared with other *Pectobacterium* strains; these features were most likely acquired via horizontal gene transfer. This work indicates that *P. wasabiae* has been present, though unnoticed, in European and maybe in American potato fields for a long time. Genome analysis is supplemented with experimental results to suggest novel virulence determinants of *Pectobacterium*; many of these determinants could be important during the poorly characterized latent stage of infection.

## Results/Discussion

### Phylogenetic analysis identifies SCC3193 as *Pectobacterium wasabiae*

The species status of *Pectobacterium* sp. SCC3193 was questioned after an initial review of the recently sequenced genome of SCC3193 by our *Pectobacterium* sequencing consortium in Helsinki, Finland (CP003415, Koskinen *et al.*, in press). We discovered distinctive sequence similarity of SCC3193 to the strain WPP163 (NC_013421.1), which was sequenced in 2009 by another group (Nicole Perna and coworkers, and US DOE Joint Genome Institute; unpublished). WPP163 was isolated from potato stem and classified as *P. wasabiae* prior to the genome sequencing [34]. Originally, *Pectobacterium* sp. SCC3193 was identified as *Pectobacterium carotovorum* based on disease symptoms in potato, the ability to produce PCWDEs, fatty acid composition and other biochemical properties. Subsequent studies suggested that SCC3193 may not be a typical *P. carotovorum* strain due to its LPS composition, sensitivity to T4 phage, decreased ability to macerate plant tissues and inability to grow at +37°C [23]. However, *P. carotovorum* has been recognized as a highly variable species that is composed of several subspecies. Thus, differences in phenotype have been accepted [3]. At the time of the isolation and characterization of SCC3193, the species *Pectobacterium wasabiae* (previously called *Erwinia carotovora* subsp. *wasabiae*) had not yet been described. The type strain (CFBP 3304^T^) of *P. wasabiae* was isolated from wasabi (Japanese horseradish) in 1987 [3], [35]. Reports of *P. wasabiae* isolates are limited when compared to the number of *P. carotovorum* and *P. atrosepticum* reports [35]–[40]. To evaluate the species status of SCC3193, we conducted a thorough phylogenetic analysis of SCC3193, which included biochemical and genomic methods. The genome of *P. wasabiae* CFPB 3304T was sequenced by our consortium to be used as a reference in this phylogenetic analysis.

#### A phylogenetic tree indicates that SCC3193 is *Pectobacterium wasabiae*

**Figure 1:**
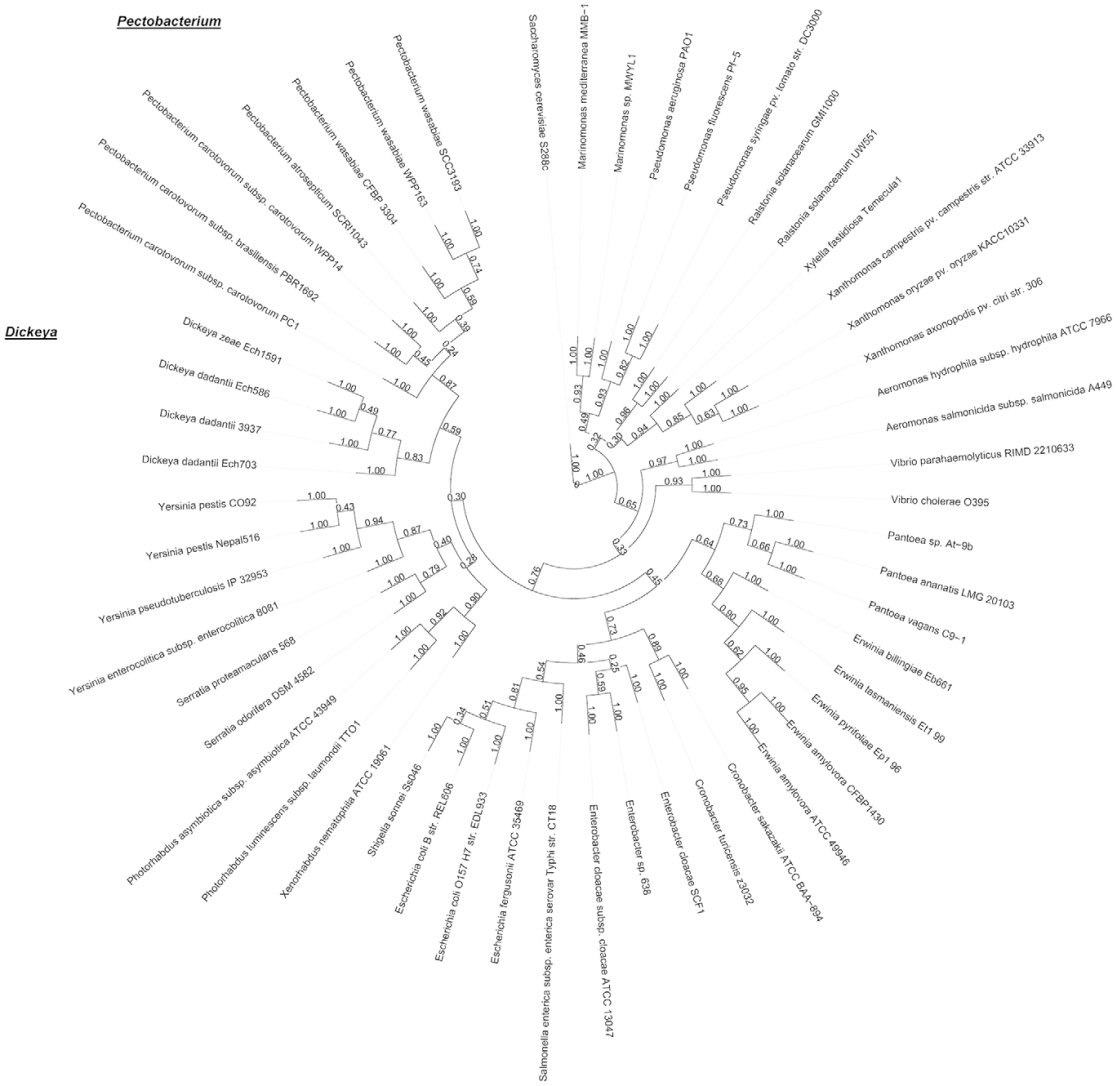
Phylogenetic tree constructed of 51 orthologous protein groups from 53 bacterial strains and yeast as an outgroup. For the selection of the orthologous groups, reciprocal best hits were determined based on similarities detected using the fast protein sequence database search tool SANS. The ortholog groups were aligned using Muscle and bootstrapped trees were created with RAxML. Consensus tree was built using Consense and visualized with iTOL. The values presented in the figure indicate bootstrap values calculated based on 51000 bootstrapped trees. The *Pectobacterium* and *Dickeya* groups are highlighted.

We first performed a brief set of standard biochemical tests, which are commonly used to distinguish *P. wasabiae* from *P. carotovorum*, to determine the species of SCC3193. These tests could not unambiguously place SCC3193 into any of the known species underlining previous difficulties to determine the taxon. SCC3193, as well as the *P. wasabiae* type strain (CFBP 3304^T^), differed from the *P. carotovorum* type strain (CFBP 2046^T^) with respect to growth in 5% NaCl and the ability to grow at +37°C and from the *P. atrosepticum* type strain (HAMBI 1429^T^) with respect to growth in 5% NaCl, production of reducing sugars and fermentation of α-methyl-glucoside. However, SCC3193 also differed from the *P. wasabiae* type strain (CFBP 3304^T^) in utilization of melibiose and raffinose. Subsequently, we utilized the genomic information for the taxonomic characterization of SCC3193. *Pectobacterium* sp. SCC3193 and *P. wasabiae* CFBP 3304^T^, which was sequenced for this purpose (AKVS00000000), and 52 additional reference species were compared in an extended multilocus sequence analysis (51 loci) and used for phylogenetic analysis. For the selection of the 51 orthologous groups (Dataset S1), reciprocal best hits were determined based on similarities detected using the fast protein sequence database search tool SANS [41]. For the 51 orthologous groups, we created multiple alignments using Muscle and bootstrapped them using RAxML. All the bootstrapped trees were merged to generate a phylogenetic tree using the Consense program in the Phylip package. In this analysis, SCC3193 was grouped with *P. wasabiae* WPP163 and *P. wasabiae* CFBP 3304^T^. The *P. wasabiae* clade including SCC3193 was also close to *P. atrosepticum* SCRI1043. Notably, SCC3193 was placed in a separate clade from *P. carotovorum* strains (Figure 1). This finding indicates that SCC3193, WPP163 and the *P. wasabiae* type strain form an evolutionary group distinct from other *Pectobacterium* species. The phylogenetic tree is in agreement with previous reports regarding the taxonomy of the soft rot enterobacteria *Pectobacterium* and *Dickeya* [2], [3], [36], [42], further supporting our results. In conclusion, our phylogenetic analysis of SCC3193 resulted in the novel finding that SCC3193 does not belong to the species *P. carotovorum* but to a completely different species, *P. wasabiae*.

#### The proteome comparison of soft rot bacteria confirmed the species of SCC3193

**Figure 2:**
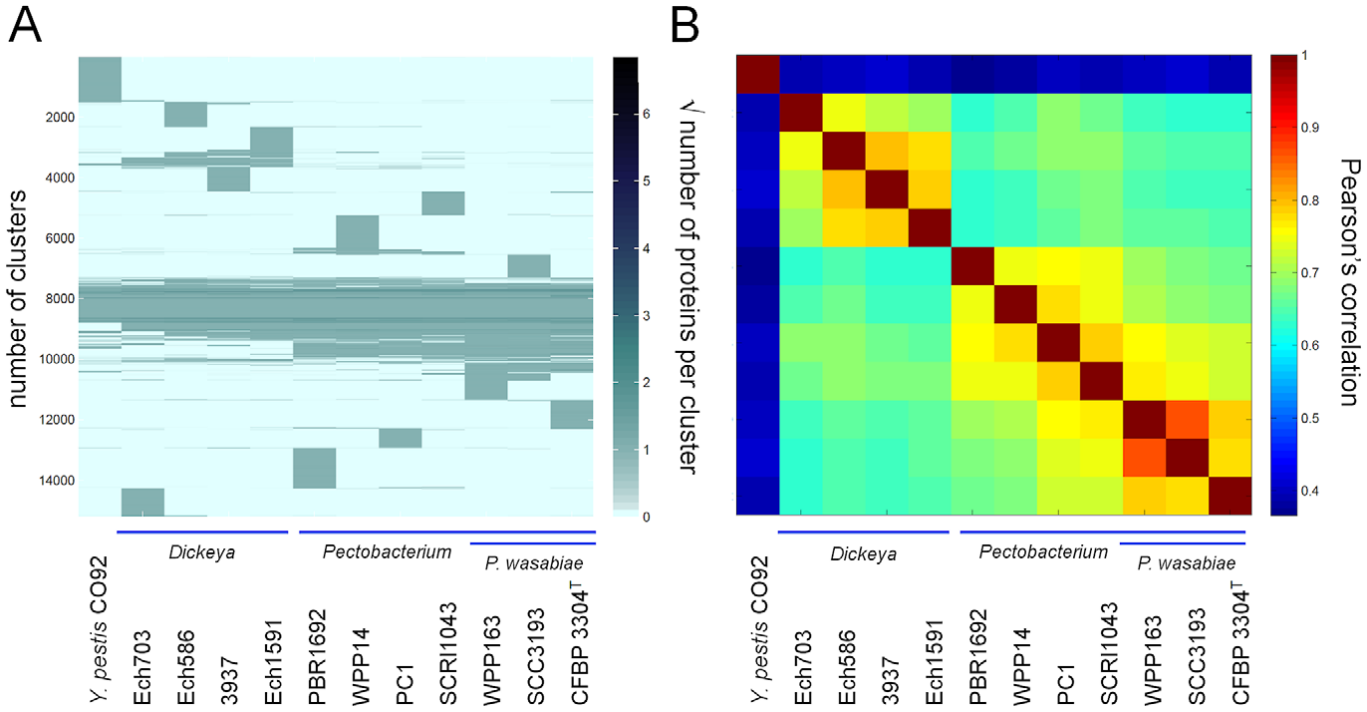
Comparison of proteomes of *Pectobacterium* and *Dickeya* strains. The strain numbers correspond to the following species: *Yersinia pestis* CO92 (outgroup), *Dickeya dadantii* Ech703, *Dickeya dadantii* Ech586, *Dickeya dadantii* 3937, *Dickeya zeae* Ech1591, *Pectobacterium carotovorum* subsp. *brasiliensis* PBR1692, *Pectobacterium carotovorum* WPP14, *Pectobacterium carotovorum* subsp. *carotovorum* PC1, *Pectobacterium atrosepticum* SCRI1043, *Pectobacterium wasabiae* WPP163, *Pectobacterium wasabiae* SCC3193 and *Pectobacterium wasabiae* CFBP 3304^T^. (A) OrthoMCL clusters were converted into an orthologs vs. species matrix and visualized as a heat map. The core genome is visualized in the middle of the figure, and species and strain-specific protein clusters can be found above and below the core. (B) The correlations between proteomes were calculated and visualized to indicate their phylogenetic relationships.

We applied a novel approach to find evolutionarily relevant groups within the soft rot bacteria and confirm the species of SCC3193. We compared proteomes using clustering with OrthoMCL and visualized them on a heat map. In the heat map, core genome, strain, species and genus-specific protein clusters are clearly visible; the heat map also groups phylogenetic clades together (Figure 2A). The correlations of all strains were calculated based on proteomes (Figure 2B), and they were in agreement with the phylogenetic clades of *Pectobacterium* and *Dickeya* (Figure 1). The comparison of all the sequenced *Pectobacterium* and *Dickeya* species with *Yersinia pestis* CO92 as an outgroup showed that the correlation between SCC3193 and *P. wasabiae* WPP163 was approximately 0.9 (bright red squares in Figure 2B). The correlation of SCC3193 and WPP163 with the type strain of *P. wasabiae* was also high. The high correlation of proteomes may indicate a close evolutionary relationship, suggesting that SCC3193 and WPP163 belong to the same species, which is most likely *P. wasabiae*.

The proteome comparison approach is a novel and simple tool for the characterization of new isolates and the revision of previous phylogenetic taxa. Clustering may also work as a tool for detecting species or group-specific proteins and investigating functional differences between strains. However, we found that the use of different ORF predictions for different strains may result in a relatively high error rate; therefore, we propose that ORF predictions need to be unified before the clustering of proteomes.

#### SCC3193 has nearly complete synteny with *Pectobacterium wasabiae* WPP163

**Figure 3:**
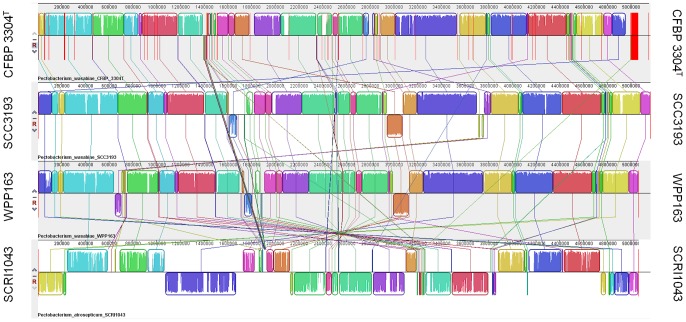
Synteny of *Pectobacterium wasabiae* and *Pectobacterium atrosepticum* genomes. Pairwise alignments of genomes were generated using Mauve. *P. wasabiae* CFBP 3304^T^ contigs were aligned according to *P. wasabiae* SCC3193. The sequence similarity in the pairwise alignment of *P. wasabiae* SCC3193 and CFBP 3304^T^ was 80.6%. The similarity between SCC3193 and WPP163 was 92.6% and between WPP163 and CFBP 3304^T^ was 80.9%. The sequence similarity compared to *P. atrosepticum* was 71.3% in the case of SCC3193, 69.6% for CFBP 3304^T^ and 72.2% for WPP163.

To investigate the relationships among all sequenced *P. wasabiae* strains isolated from different continents and host crops (SCC3193 from potato in Europe, WPP163 from potato in North-America and CFBP 3304^T^ from Japanese horseradish in Asia), we compared their genome sequences to each other and to the closest neighbor in the clade (*P. atrosepticum* SCRI1043) using Mauve, which is a multiple genome alignment tool. SCC3193 shows almost complete synteny with *P. wasabiae* WPP163 and differs by only 7.4% in the pairwise alignment (pairwise genome content distance) (Figure 3). Because the genome of the type strain is in contigs, it was aligned according to SCC3193. The type strain differs by 19.4% and by 19.1% from SCC3193 and WPP163, respectively. The differences between *P. wasabiae* strains in the pairwise alignments are much smaller than the differences between *P. wasabiae* strains and *P. atrosepticum*; SCC3193 differs 28.7%, WPP163 differs 27.8% and CFBP 3304^T^ differs 30.4% from *P. atrosepticum* SCRI1043. Taken together, the results of the pairwise alignment support the notion that SCC3193, WPP163 and CFBP 3304^T^ belong to the same species. This is most evident for SCC3193 and WPP163, due to their almost complete synteny. However, we cannot rule out the possibility that SCC3193 and WPP163 could represent a novel potato-related subspecies of *P. wasabiae*, a species that has previously been found mainly from Japanese horseradish. This possibility is especially notable when we consider the small number of *P. wasabiae* isolates characterized and the relatively large difference between the type strain and SCC3193/WPP163 compared with the small difference between SCC3193 and WPP163. Based on the current knowledge, we suggest that these three strains are best classified as *P. wasabiae*.

#### *Pectobacterium wasabiae* may have been present but unnoticed on potato fields for a long time

Our work and the very recent work of Nabhan and colleagues at the end of 2011 [40] suggest that some *P. wasabiae* strains have been misidentified as *P. carotovorum* in the past. Nabhan et al. [40] showed that three *P. carotovorum* strains isolated from Europe can now be classified as *P. wasabiae* based on multilocus sequence alignment. At this point, it is unclear whether *P. wasabiae* is an emerging pathogen in potato fields or if the rising number of *P. wasabiae* reports [36], [37], [39] is a consequence of improved DNA sequence-based characterization methods. Our genomic approach and comparison of SCC3193 to the type strain of *P. wasabiae* could provide a solid basis for further investigation of the distribution and phylogeny of *P. wasabiae*.

### *Pectobacterium wasabiae* SCC3193 has an arsenal of established virulence determinants

To obtain an overall view of SCC3193 virulence factors, we mined the genome for known virulence determinants of *Pectobacterium*. Because we are working with an established model strain, many virulence-associated determinants of SCC3193 have already been identified in genetic studies, and some of these were even originally described in SCC3193. This may create a bias that is reflected in the number of published virulence-associated genes found in the genome of SCC3193.

#### Plant cell wall-degrading enzymes are essential for pathogenesis of *Pectobacterium*

The production of PCWDEs is the hallmark of soft rot pectobacteria. We identified a total of 39 known or putative pectinases, cellulases and proteinases (Table S1). Most of the PCWDE genes are shared by all sequenced *Pectobacterium* strains, with the exception of several genes for putative proteinases, of which two may be specific to *P. wasabiae*, and one is present only in SCC3193 and the type strain. The number of strain-specific proteinases is similar in all the compared strains. PehK, HrpW and a putative pectate lyase are not present in *P. wasabiae* strains, although they are present in the other compared *Pectobacterium* strains. *P. carotovorum* subsp. *carotovorum* PC1 has one additional putative polygalacturonase. Our GO term based proteinase analysis is supplemented with previously described putative proteinases that are not present in *E. coli* strains; therefore, they may represent a novel proteinase class associated with plant pathogens [22]. One previous report indicates that potato isolates of *P. wasabiae* are less virulent than *P. carotovorum* strains [34]. In our observation, we have also found that SCC3193 has a lower capacity to macerate potato tubers than *P. atrosepticum* SCRI1043 and *P. carotovorum* SCC1. It remains unclear whether the lack of PehK, HrpW and the pectate lyase present in other *Pectobacterium* has an effect on the virulence of *P. wasabiae*.

#### Previously characterized virulence determinants of *Pectobacterium* present in SCC3193 and other *P. wasabiae* strains with sequenced genome

The flagella-encoding cluster (W5S_1760–W5S_1810) is most likely elementary to the virulent lifestyle [24], [43], [44] and can be found in the genomes of all *P. wasabiae* strains. The enterobacterial common antigen (ECA) encoding cluster (W5S_4355–W5S_4365) and the LPS cluster (W5S_4520–W5S_4538) are present in all *P. wasabiae* strains as well. Mutagenesis of the *rffG* (ECA and LPS core) and *waaJ* (LPS) genes reduces the virulence of *P. atrosepticum* in potato [12], [45]. All *P. wasabiae* strains also harbor the 3-hydroxy-2-butanone pathway (*budRAB*; W5S_0740–W5S_0742 and *budC*; W5S_0317) that was recently shown to have an effect on the alkalization of the environment, which is hypothesized to be a partial reason for the decreased virulence of the *budB* mutant in potato compared with the wild-type *P. carotovorum* [19]. *P. wasabiae* and other *Pectobacterium* species contain a few virulence factors encoded by single genes. One is the necrosis-inducing virulence protein (Nip; W5S_1316), which has been experimentally demonstrated in *P. wasabiae* SCC3193, *P. atrosepticum* SCRI1043 and *P. carotovorum* ATTn10 to be a virulence determinant [15], [46]. Another single gene encoding a putative virulence factor (*svx*; W5S_0937) is highly similar to a secreted avirulence factor gene in *Xanthomonas*, and it has been shown to contribute to the virulence of *P. atrosepticum* [16]. The citrate transporter, which is proposed to enhance the colonization of *P. atrosepticum* in potato tubers by decreasing citrate concentration [18], was also found in *P. wasabiae* strains SCC3193 (W5S_4105), WPP163 and CFBP 3304^T^.

#### Virulence is regulated via a complex network

The regulation of virulence in *Pectobacterium* has been extensively studied, and a large number of regulators controlling pathogenicity have been characterized both genetically and through molecular studies [11]. SCC3193 has been one of the main models utilized to elucidate virulence regulation in pectobacteria, and as a result, a number of regulatory mutants affecting virulence and PCWDE production have been characterized. The complex regulatory network in SCC3193 is relatively well described and includes the following: a cell density-dependent quorum sensing system, first described in SCC3193 (ExpI; W5S_4607, ExpR; W5S_4606/W5S_1749, LuxS; W5S_1019); several two-component systems involved in sensing the environment (ExpAS; W5S_1457/W5S_3687, PehRS; W5S_2096/W5S_2095, PmrAB; W5S_4173/W5S_4174); the Rcs phosphorelay system (RcsC; W5S_3208, RcsD; W5S_3206, RcsB; W5S_3207); a few global regulators (RsmA; W5S_1009, KdgR; W5S_2118, ExpM; W5S_2224, Hor; W5S_2637); and a regulatory RNA (rsmB; 3645019–3644673) [7]–[9], [17], [27], [30], [32], [33], [47]–[50]. All the genes encoding the above mentioned components of the regulatory network are also present in the genomes of *P. wasabiae* WPP163 and CFBP 3304^T^. In addition to these previously characterized regulators of virulence, we identified a number of additional putative regulators in the genome of SCC3193 (Broberg *et al.*, in preparation) both from the core genome, often conserved among enterobacteria, and from the genomic islands, which may represent novel regulators or regulators co-opted for species- or niche-specific interactions.

#### Secretion systems pass virulence determinants across the bacterial cell wall from cytosol to environment

The delivery of virulence determinants into the host is typically a central feature in pathogenesis. In pectobacteria, pectinases and cellulases are secreted through a type II secretion system (T2SS, W5S_1291–W5S_1305) that is also called the Out-system, and its inactivation renders *Pectobacterium* avirulent [24], [51]. Proteinases are usually secreted through a type I secretion system (T1SS), and it has been shown that PrtW (W5S_2894) contributes to virulence in SCC3193 [28]. In SCC3193, a virB-type IV secretion system (T4SS, W5S_1616–W5S_1624), which is best known as the Ti-plasmid transferring system of *A. tumefaciens*, is similar to that of *P. atrosepticum* [13]. A similar T4SS is also present in *P. wasabiae* CFBP 3304^T^, but we were not able to find it from *P. wasabiae* WPP163 on protein or nucleotide level. Interestingly, the similar T4SS cluster is also present in *P. carotovorum* subsp. *brasiliensis* but not in *P. carotovorum* subsp. *carotovorum* WPP14 or PC1. Furthermore, *P. wasabiae* has a T6SS that was previously characterized in *P. atrosepticum* as a virulence determinant [14]. Intriguingly, SCC3193 harbors two T6SS clusters (W5S_0962–W5S_0978 and W5S_2418–W5S_2441) and this is also the case for strains WPP163 and CFBP 3304^T^. Additionally, SCC3193 has genes for 26 haemolysin co-regulated proteins (Hcp) or valine-glycine repeat protein G (VgrG) proteins that may be related to the function of T6SS. The number of Hcp and VgrG encoding genes varies among bacterial species and even strains: *P. wasabiae* WPP163 carries 18 and *P. wasabiae* CFBP 3304^T^ eight *hcp* or *vgrG* genes. The actual function of these proteins is not fully understood; they may have a role in the construction of the syringe-like secretion machinery of T6SS, or they may act as effectors [52]. The roles of the T6SS among Gram-negative bacteria appear to be diverse. Depending on the type of effectors delivered and possibly on the structure of the syringe, T6SS targets several organisms, including humans, other animals, plants and bacteria [52]–[54].

### *Pectobacterium wasabiae* lacks type III secretion system

Only a few previously described virulence-related genes in *Pectobacterium* were not found in SCC3193: namely, the genes for coronafacic acid synthesis (specific to *P. atrosepticum*) and the type III secretion system (T3SS) present in many *P. atrosepticum* and *P. carotovorum* strains [11], [13]. The T3SS is composed of the injection machinery encoded by the conserved *hrp*/*hrc* gene cluster and of a species/strain-specific collection of effectors required to suppress the basal defenses of the host [55]. Analysis of the SCC3193, *P. wasabiae* WPP163 and the type strain (CFBP 3304^T^) genomes failed to identify any traces of T3SS. It appears typical of *P. wasabiae* that it lacks this widespread and important virulence determinant. These results are in agreement with previous failed efforts to detect T3SS in SCC3193 or other *P. wasabiae* strains; for example, no signs of this injection machinery or the associated effectors (encoded by *hrpN*, *dspE* and *hecB* genes) have been found [34], [36], [56]. Although absent from *P. wasabiae*, T3SS contributes to the virulence of other soft rot species [57]–[59].

Contrary to hemibiotrophic pathogens such as *Pseudomonas syringae*, where T3SS is essential for pathogenicity [21], the role of T3SS in necrotrophic soft rot bacteria appears quite complex. T3SS contributes to the virulence of *P. carotovorum*, *P. atrosepticum* and *Dickeya* strains, but even strains naturally lacking T3SS, such as *P. wasabiae*, are still able to infect potatoes. A recent study showed no clear correlation between virulence and the presence of T3SS in *P. carotovorum* [34]. The relatively modest significance of T3SS to pathogenicity in pectobacteria is also reflected in the small number of T3 effectors found in these species compared, for example, to the dozens of known effectors of *Pseudomonas syringae* [60]. Thus, *Pectobacterium* may have alternative ways of modifying the host plant at the initiation of infection.

The lack of T3SS and numerous effectors may also benefit *Pectobacterium* by widening its host range. Soft rot bacteria, excluding the potato-specific *P. atrosepticum*, are often found to colonize a wide range of food crops and ornamental plants, while many T3SS-dependent plant pathogens, such as *Erwinia*, *Pseudomonas*, *Xanthomonas* and *Xylella*, have a very narrow host range. It is well documented that some T3 effectors may act as colonization-inhibiting avirulence proteins that are recognized by the plant [55]. Virulence assays to investigate the role of T3SS in soft rot bacteria in planta thus far indicate that in a complex natural niche, T3SS may have an important role under certain conditions but not in others.

### Genomic islands have a *Pectobacterium wasabiae* twist

**Figure 4:**
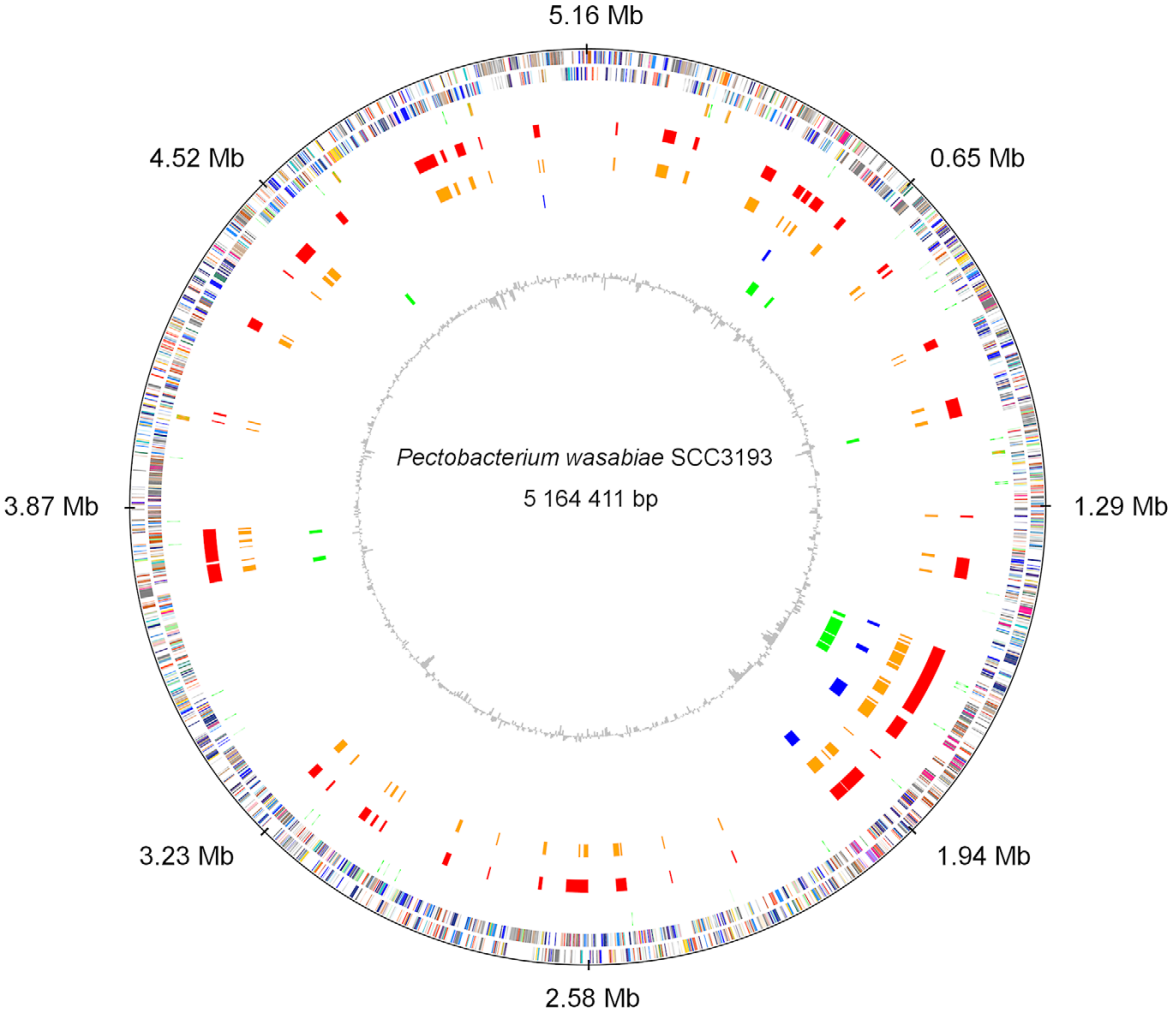
Circular representation of the chromosome of *Pectobacterium wasabiae* SCC3193. The circles from outer to inner represent open reading frames on both strands, tRNAs (green) and rRNAs (orange), manually curated genomic islands (red), SIGI-HMM predicted islands (orange), IslandPath-DIMOB predicted islands (blue), IslandPick predicted islands (green) and GC percentage (gray).

Horizontal gene transfer plays an important role in the evolution of bacteria. It enables the rapid acquisition of beneficial traits in a single event. These gene clusters of probable horizontal origin are termed genomic islands (GIs/GEIs) or horizontally acquired islands (HAIs) [11], [61], [62]. To characterize the genome composition of SCC3193 and identify horizontally acquired virulence determinants or other adaptive traits, we determined putative GIs in the genome of SCC3193. We used two sequence composition-based GI prediction methods, SIGI-HMM and IslandPath-DIMOB [63], [64], which were found to have the highest overall accuracy of the six methods tested in a recent bioinformatic study [65]. In addition, we used one comparative genomic-based GI prediction method, IslandPick [65]. The three methods predicted a different number of islands and smaller islets: six for IslandPath-DIMOB, ten for IslandPick and over hundred for SIGI-HMM (63 consisted of five or more successive ORFs) (Figure 4). Automated predictions were subsequently manually curated, resulting in a total of 56 genomic islands (Table S2). The GIs comprise ∼0.86 Mb, which is 16.7% of the size of the genome and encompasses 21.6% of all ORFs (1040 of the 4804 ORFs). In comparison, 17.6% of the ORFs of *Escherichia coli* MG1655 were estimated to have been acquired horizontally [66]. In general, GIs are estimated to comprise between 1.6% and 32.6% of the ORFs in prokaryotic genomes [67].

A majority of the SCC3193 islands can be found identically or with slight permutations in *P. wasabiae* WPP163, whereas less than half of the islands are present in the genome of *P. wasabiae* CFBP 3304^T^ (Table S2). Approximately 15 islands were specific to *P. wasabiae* among the soft rot group. Some *Pectobacterium*-specific islands (for example, GI_44) or islands partially present in other *Pectobacterium* strains but completely lacking from *Dickeya* (for example GI_13 and GI_36) were also discovered. A number of islands were specific to SCC3193 and could not be found in any other strains of soft rot bacteria (for example, GI_20, GI_40, GI_43 and GI_54). Furthermore, a few islands showed a significant similarity to the genomes of bacteria outside the soft rot species (GI_9, GI_10, GI_30, GI_40, GI_44 and GI_50). Functional predictions for the genes on the islands in SCC3193 suggested the presence of a high number of mobile element genes and genes with unknown functions. The islands were also found to contain several genes for known virulence determinants, such as Nip (GI_17), which is necessary for the full virulence of SCC3193 [15], and DsbA (GI_56), which is required for the correct conformation of many secreted virulence proteins in *P. atrosepticum* and *P. carotovorum* [68], [69]. Most of the islands were shown to carry genes with predicted functions that could often be potentially associated with plant colonization or virulence (Table 1, Table S2, Figure 4).

**Table 1:** *Pectobacterium wasabiae* SCC3193 genomic islands of 10 kb or larger and selected smaller islands.

GI	Locus tags	Size (bp)	ORFs	Predicted function
**1**	W5S_0053–W5S_0061	5087	9	Type VI secretion-related Hcp
**2**	W5S_0140–W5S_0169	27368	30	Rhs element related genes and YD repeats of unknown function
**3**	W5S_0206–W5S_0219	9091	14	Ribosomal proteins and tRNA
**4**	W5S_0358–W5S_0389	26510	32	Rhs element-related genes and YD repeats of unknown function
**5**	W5S_0444–W5S_0452	13875	9	Contact-dependent growth inhibition (Cdi)
**6**	W5S_0459–W5S_0472	13237	14	Unknown function (part of Vic1)
**7**	W5S_0477–W5S_0498	22345	22	Lipoprotein transportation system, HopL1 similar to *P. syringae* (Vic2)
**8**	W5S_0538–W5S_0556	12861	19	Rhs element-related genes of unknown function
**17**	W5S_1313–W5S_1319	5821	7	Necrosis-inducing protein (Nip)
**18**	W5S_1461–W5S_1470	13243	10	Rhs element-related genes of unknown function
**19**	W5S_1471–W5S_1540	67603	70	Unknown function
**20**	W5S_1543–W5S_1611	77629	69	Pilus/fimbrial system, carocin-like bacteriocin
**22**	W5S_1635–W5S_1679	38721	45	Unknown function
**24**	W5S_1761–W5S_1810	47778	50	Flagella
**25**	W5S_1811–W5S_1848	29762	37	Unknown function
**28**	W5S_2307–W5S_2350	28485	44	Unknown function
**29**	W5S_2396–W5S_2407	11110	12	Rhs element-related genes of unknown function
**30**	W5S_2416–W5S_2441	29426	26	Type VI secretion (T6SS-2)
**33**	W5S_2670–W5S_2679	12375	10	Type VI secretion-related genes
**34**	W5S_2819–W5S_2826	4194	8	Pyocin/colicin resistance
**35**	W5S_2846–W5S_2854	6565	9	Benzoic acid/salicylic acid methyltransferase
**36**	W5S_2866–W5S_2889	17874	24	Phage tail-like bacteriocin
**38**	W5S_3001–W5S_3022	18277	22	Lipopolysaccharide (LPS)
**39**	W5S_3422–W5S_3455	29251	34	Arsenate reductase
**40**	W5S_3465–W5S_3482	13686	18	Resistance to arsenate (*ars*)
**42**	W5S_3517–W5S_3565	38225	49	Type IV pili (*pil*)
**44**	W5S_3785–W5S_3791	3983	7	Membrane proteins
**45**	W5S_3956–W5S_3968	12158	13	Rhs element-related genes of unknown function
**48**	W5S_4116–W5S_4128	10532	13	rRNA and tRNA
**49**	W5S_4135–W5S_4171	21262	37	Ribosomal proteins and tRNA
**50**	W5S_4247–W5S_4262	15097	16	Bacterial microcompartment of unknown function
**51**	W5S_4435–W5S_4475	32025	40	Unknown function
**53**	W5S_4520–W5S_4538	18358	19	Lipo-oligo/polysaccharide (LOS/LPS)
**54**	W5S_4558–W5S_4587	20415	30	Unknown function
**55**	W5S_4688–W5S_4695	3239	8	Plant ferredoxin-like protein FerE
**56**	W5S_4700–W5S_4704	3286	5	Disulphide bond formation on secreted proteins, including plant cell wall-degrading pectinases and proteinases (DsbA)

#### Plant ferredoxin-like protein and benzoic acid/salicylic acid carboxyl methyltransferase may have a eukaryotic origin

Two genes of possible eukaryotic origin were identified in the genome of SCC3193, and they were also present in the genome of *P. wasabiae* WPP163. These genes appear to be unique to potato isolates of *P. wasabiae*, as similar genes are not present in the genome of the *P. wasabiae* type strain CFBP 3304^T^ isolated from Japanese horseradish or in the genomes of any other bacterial species characterized, and the deduced proteins are most similar to plant proteins. One of these genes encodes the plant ferredoxin-like protein FerE (W5S_4691, GI_55), which has been found to enhance oxidative stress tolerance and have an effect on the fitness of SCC3193 in planta [17]. The other gene encodes a putative S-adenosyl-L-methionine∶benzoic acid/salicylic acid carboxyl methyltransferase (W5S_2852, GI_35). The corresponding enzymes in plants, which methylate benzoic acid and salicylic acid, participate in plant defense responses and the biosynthesis of floral scents [70], [71]. Methyl salicylate, produced by methylation of salicylic acid in response to pathogen attack, is thought to act as a mobile signal for systemic acquired resistance, which provides broad-spectrum resistance against further pathogen attacks throughout the plant [71]. The overexpression of plant salicylic acid methyltransferase has been shown to affect plant defense responses and pathogen resistance [72], [73]. This finding suggests the intriguing possibility that the *P. wasabiae* protein may be used to modify salicylic acid-dependent defenses and could represent a novel mechanism for a plant pathogen to manipulate its host. The ability to manipulate the induction of defense responses could be of great advantage to the T3SS-deficient *P. wasabiae* at the beginning of the infection, enabling it to colonize the host without being confronted with an arsenal of defenses triggered by plant recognition of the conserved microbe-associated molecular patterns (MAMPs).

#### A novel bacterial microcompartment is present in *Pectobacterium wasabiae*

We found a novel bacterial microcompartment (BMC) cluster (GI_50) present only in *P. wasabiae* strains (SCC3193, WPP163 and CFBP 3304^T^) and a handful of non-soft rot bacterial species. Bacterial microcompartments are enclosed protein complexes that encapsulate the sequential reaction steps for selected metabolic pathways, and they are thought to be horizontally transferring genomic elements [74]–[76]. Thus far, the best-known BMCs are CO_2_-fixing carboxysome, propanediol utilization (Pdu) BMC and ethanolamine utilization (Eut) BMC. Additional findings include the pyruvate-to-ethanol pathway in *Vibrio furnissii* and the ethanol-to-acetate pathway, together with the separate BMC for glycerol metabolism in *Clostridium kluyveri* [76], although these results have not yet been fully investigated. The BMC of SCC3193 is similar to the pyruvate-to-ethanol BMC of *V. furnissii*; this is based on the core enzyme pyruvate formate-lyase. However, the SCC3193 cluster includes an additional acetate kinase that is not present in any of the described microcompartment clusters. In *Salmonella*, it is thought that a household acetate kinase participates in the Eut pathway [77]. The rest of the genes in the cluster are typical components of Eut/Pdu BMCs, which mainly encodes the microcompartment multiprotein complex.

The BMC cluster in *P. wasabiae*, with a small variation in synteny, is present in the following strains with complete genomes: *Escherichia coli* CFT073 (c4524–c4538 and c4545–c4548), *Rhodospirillum rubrum* ATCC11170 (Rru_A0902–Rru_A0919), *Rhodopseudomonas palustris* BisB18 (RPC_1163–RPC_1179), *Rhodobacter capsulatus* SB1003 (RCAP_rcc02196–RCAP_rcc02214), *Clostridium beijerinckii* NCIMB 8052 (Cbei_4050–Cbei_4065) and *Shewanella* sp. W3-18-1 (SputW3181_0417–SputW3181_0429). These bacteria are anaerobes or facultative anaerobes with extremely versatile metabolic systems [78]–[86]. It is not clear what this microcompartment does in *P. wasabiae* strains, but we speculate that it enhances the anaerobic utilization of different carbon sources. It is well known that *Pectobacterium* can grow in anaerobic conditions, and it is possible that this BMC provides an advantage in the utilization of carbon for *P. wasabiae* when compared to other soft rot bacteria.

#### Liposaccharide-encoding clusters differ from *Pectobacterium carotovorum*

Previously, it was shown that *P. wasabiae* SCC3193 has a different LPS composition than *P. carotovorum* strains [23]. Two novel putative liposaccharide clusters were found in SCC3193. These clusters localized to GI_38 and GI_53; the first cluster is found only in potato isolates of *P. wasabiae* (SCC3193, WPP163), with the exception of the first three genes (*rfbA*, *rfbC* and *rfbD*), which are commonly present in *Pectobacterium*. This previously unknown cluster contains features of the clusters that encode LPS, capsular polysaccharides and lipo-oligosaccharide (LOS). The second cluster is present in potato isolates of *P. wasabiae* and in *P. atrosepticum* (*waa* cluster), and it is partially present in other *Pectobacterium* strains including the *P. wasabiae* type strain CFBP 3304^T^. These findings likely explain the differences found between the LPS composition of SCC3193 and that of *P. carotovorum*. Lipopolysaccharides and similar structures on the surface of bacteria have several roles in plant-microbe interactions. They may enhance the attachment of bacteria to the plant tissue, protect the bacteria from plant-derived toxins and be recognized by the plant, which may lead to a defense response or to a mutualistic lifestyle [87]. The variation observed in the LPS composition within *Pectobacterium* may indicate an adaptation to different environmental conditions.

#### Arsenic resistance may have a recent origin

SCC3193 was shown to harbor an arsenic resistance cluster. Arsenic resistance genes (*ars*) are widespread among bacteria, and they can be plasmid-borne or chromosomal [88]. SCC3193 GI_40 harbors an arsenic resistance cluster (6303 bp). The *ars* cluster was originally located adjacent to an SIGI-HMM predicted small (3133 bp) island, but we manually expanded the island to cover the cluster as well. The novel island (13686 bp) showed significant similarity to the genome of *Enterobacter cloacae* subsp. *cloacae* ATCC 13047 (blastn query coverage 87%, E 0.0). This region is missing from the genomes of other *Pectobacterium* strains. However, less similar Ars proteins are present in *P. atrosepticum* SCRI1043 (ECA1603–ECA1606, HAI7) and *P. carotovorum* WPP14 (GI:227326266, GI:227326267, GI:227326268). Evolutionary studies suggest that the exchange of *ars* clusters between plasmids and chromosomes and the horizontal transfer of the cluster are frequent [89]. Therefore, it appears likely that the *ars* clusters of SCC3193 and *E. cloacae* share a relatively recent common origin that is different from the clusters of *P. atrosepticum* SCRI1043 and *P. carotovorum* WPP14. Other *Pectobacterium* strains, including *P. wasabiae* WPP163 and CFBP 3304^T^, contain only putative arsenate reductase (*arsC*) and lack other components. SCC3193 also has two additional copies of *arsC* (W5S_3181 and W5S_3448 on GI_39). SCC3193 was isolated from southern Finland, where the highest arsenic concentrations of the cultivated soils in Finland are found [90]. The arsenic resistance cluster may have been an advantage under these environmental conditions. It is likely that arsenic resistance genes benefit all *Pectobacterium* species in the environment and inside plants that grow in soils containing arsenic.

#### Rhs elements are often related to outer membrane structures

A number of Rhs (recombination hot-spot) element genes were found in the genomic islands of SCC3193 (GI_2, GI_4, GI_8, GI_18, GI_29 and GI_45) and were partially or completely missing from other soft rot strains, except GI_8 which is present with 100% query coverage and GI_29 which is present with 93% query coverage in *P. wasabiae* WPP163 (Table S2). Rhs elements comprise a variable number of core *rhs* genes from different families and extensions to the core. In addition, they contain adjacent *vgrG* and *hcp* genes and an H-rpt insertion sequence [91]. Based on early studies, Rhs elements were first thought to be mediators of chromosomal rearrangements. With an increasing number of genomic sequences available for comparative studies, the view of Rhs elements is changing, and the loci are observed as a diverse and ancient family of protein-encoding genes [92].

Rhs element genes are reported to have several roles, depending on the species. Rhs elements have been associated with T6SS, encoding its functional core proteins, namely, Hcp and VgrG [93], [94]. To identify putative T6SS-related Hcp and VgrG proteins in SCC3193, we aligned all known Hcp and VgrG proteins in SCC3193 and *P. atrosepticum*, and a crude phylogenetic analysis was performed using ClustalW2 [95], [96]. Based on this analysis, only some of the *vgrG* and *hcp* genes present in SCC3193 are related to T6SS (data not shown). *vgrG* genes have also been associated with a social behavior called self-recognition. In *Proteus mirabilis*, Hcp and VgrG protein-encoding genes form the IdsA-IdsB locus. IdsB (VgrG) is required for self-recognition [97]. Many of the Rhs elements of SCC3193 contain several YD repeat-encoding genes. Previously, Rhs-YD repeat elements have been associated with social motility in *Myxococcus xanthus* [98]. Recently, Rhs elements have also been related to a toxin/immunity system, where they play a role in the contact-dependent intercellular competition of bacteria. This system, referred to as contact-dependent growth inhibition (Cdi), is also present in *P. wasabiae* WPP163; in *D. dadantii* 3937, it has an effect on the growth of *E. coli* [99]. A similar Cdi locus is present in SCC3193 on GI_5, but it is missing from the *P. wasabiae* type strain (CFBP 3304^T^). The function of Rhs elements is not clear; based on the current evidence, Rhs, Hcp and VgrG proteins likely form bacterial cell membrane-associated complexes, where additional domains have an effect on other organisms.

#### Several fimbria/pilus systems and flagella are on genomic islands

One genomic area in particular appeared to have accumulated a number of predicted horizontally transferred genes and gene clusters (GI_18–GI_22). Of these genomic islands, GI_18–GI_21 can be found partially (>50% query coverage) also from other *P. wasabiae* strains CFPB 3304T and WPP163. However, GI_22 can be found only from *P. wasabiae* WPP163 and SCC3193 but not from CFPB 3304^T^. In addition to several predicted plasmid or phage origin clusters, this region contains putative fimbria or pilus encoding clusters, including the virB-T4SS that lies between GI_20 and GI_21. Interestingly, similar T4SS is present in *P. wasabiae* SCC3193 and CFPB 3304^T^ but not in *P. wasabiae* WPP163 pointing toward possibility that the corresponding T4SS clusters have horizontal origin or that it is deleted from WPP163. GI_18 also carries one of the putative T6SS effector encoding clusters, which is highly similar to corresponding clusters in *P. atrosepticum* (ECA2866-ECA2869 and ECA4275–ECA4278) [14], [100]. Additionally, the single copy flagellar system, which is related to the virulent lifestyle, was found on a genomic island (GI_24). The type IV pilus cluster (*pil* genes) found on GI_42 is similar to a genomic island (HAI2) in *P. atrosepticum* [11]. Similar type IV pilus is present, among soft rot bacteria, only in *P. wasabiae* SCC3193, *P. wasabiae* WPP163 and *P. atrosepticum* SCRI1043. Type IV pili mediate attachment to surfaces and twitching motility, and they are important in the pathogenesis of many animal pathogenic species in the Enterobacteriaceae family but are not known to influence virulence in *Pectobacterium* [101], [102]. Overall, very little is known about the attachment and biofilm formation of the *Pectobacterium* species. In *P. atrosepticum*, LPS is needed for full attachment on artificial surfaces [12]. In addition, *Pectobacterium* species contain several gene clusters encoding putative attachment structures, suggesting that the lack of experimental data on attachment could be due to the functional redundancy of these systems.

#### Aerobactin siderophore locus is present in potato isolates of *Pectobacterium wasabiae*

Siderophores facilitate iron acquisition from the environment. Aerobactin is a siderophore present in several enterobacterial strains. It has been characterized best from human pathogens, such as *E. coli*, *Shigella flexneri*, *Klebsiella pneumonia* and *Salmonella* sp., and the cluster is thought to be on a horizontally transferred element [103], [104]. In *P. wasabiae* SCC3193, an aerobactin synthesis cluster (W5S_0838–W5S_0842) was identified but not predicted to be located on GIs. Nevertheless, we speculate that it is a horizontally transferred element in the SCC3193 genome. An aerobactin synthesis cluster is not present in any other sequenced *Pectobacterium* except for *P. wasabiae* WPP163 (Pecwa_0947–Pecwa_0951). Previously, only one *Pectobacterium* strain (*P. carotovorum* W3C105) producing aerobactin has been identified [103]. The role of siderophores in the virulence of the *Pectobacterium* species has not been determined, but the capability of iron acquisition is an important virulence determinant for *Dickeya* [105]. Therefore, it is likely that the presence of aerobactin would contribute to the virulence of certain *P. wasabiae* strains and/or benefit the bacteria in other iron-deficient environments.

### Novel virulence determinants

The characterization of the SCC3193 genome revealed novel genes that could contribute to the virulent lifestyle. We selected a group of interesting potential virulence determinants for experimental verification. The corresponding genes were inactivated by targeted mutagenesis, and their contribution to virulence was tested on axenic tobacco seedlings and potato tuber slices. A previously characterized phytase gene in *P. wasabiae* CFBP 3304^T^ (Y17_1078) [106] is also present in *P. wasabiae* SCC3193 (W5S_4347) and *P. wasabiae* WPP163 (Pecwa_4189). In our virulence assays the phenotype of an SCC3193 phytase gene knock-out mutant was inconclusive and its potential role in virulence would require additional studies. However, the phytase gene is not unique to *P. wasabiae* as it can be found from all sequenced *Pectobacterium* strains and many other plant associated bacteria. The mutants that had phenotypes in planta were tested for their ability to grow in vitro and produce polygalacturonases and cellulases. No differences were observed between the mutants and the wild- type strain (data not shown), indicating that the phenotypes in planta are due to plant-microbe interactions and not general growth defects or a major reduction in PCWDE production.

#### SirB1 contributes to virulence of *Pectobacterium wasabiae* SCC3193

**Figure 5:**
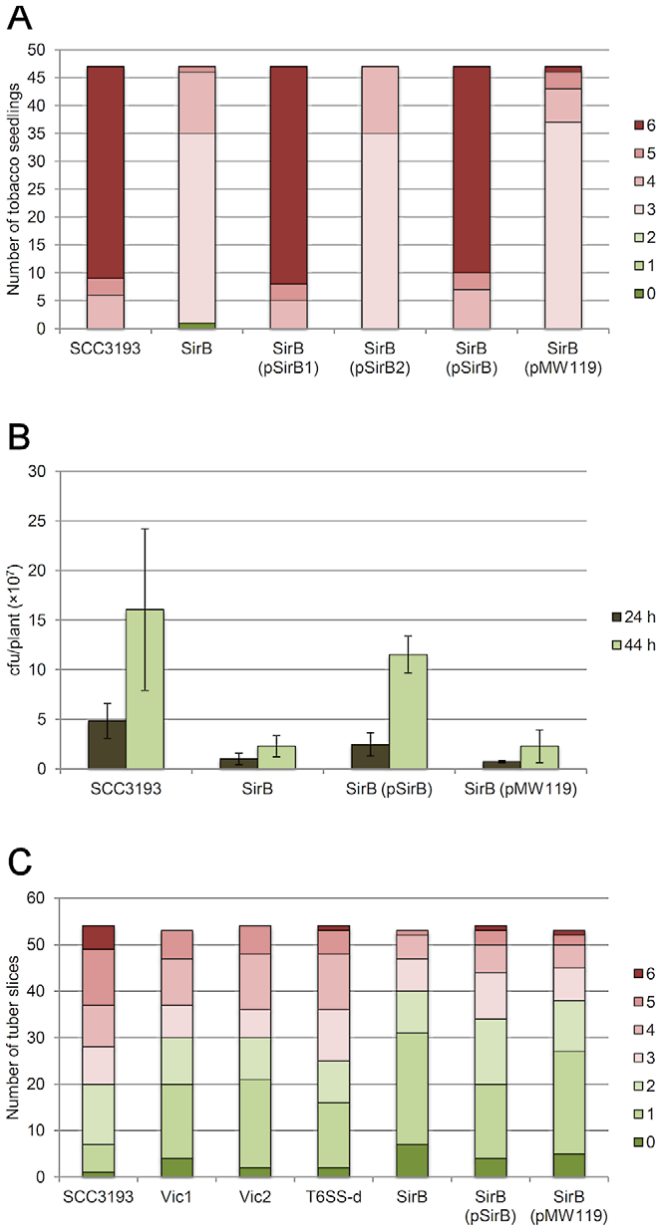
*sirB^−^*, Vic1, Vic2 and T6SS-double mutants showed decreased virulence in planta. The virulence of mutants was experimentally determined on tobacco and potato. Experiments were repeated a minimum of three times. The data from one experiment is shown for tobacco, and the combined data from five experiments are shown for potato. The strains shown are *Pectobacterium wasabiae* SCC3193, *sirB^−^*, *sirB^−^*(pSirB), *sirB^−^*(pSirB1), *sirB^−^*(pSirB2), *sirB^−^*(pMW119), Vic1, Vic2 and T6SS-double mutant. (A) Axenic tobacco seedlings (cv. Samsun) were inoculated locally with 5×10^4^ cfu, and symptoms were documented after 48 h as follows: 0 = no maceration, 1 = maceration in less than half of the inoculated leaf, 2 = maceration in more than half of the inoculated leaf, 3 = inoculated leaf was completely macerated, 4 = maceration spread to other leaves, but less than half of the plant was macerated, 5 = maceration spread to other leaves, and more than half of the plant was macerated and 6 = the whole plant was macerated. (B) To determine the bacterial growth on tobacco, samples were taken at 24 and 44 h post-inoculation, and the amount of living bacteria was measured by plate counting. For one sample, two inoculated plants were combined, and the result shows the average of 8 samples. Bars represent standard deviations. (C) Potato tuber slices (cv. Van Gogh) were inoculated with 1×10^4^ cfu, and after three days the macerated area (%) on the tuber slice was documented as follows: 0 = 0%, 1 = ∼5%, 2 = ∼25%, 3 = ∼50%, 4 = ∼75%, 5 = ∼90% and 6 = 100%.

The *sirB* locus and its surroundings are conserved within Enterobacteriaceae. SirB is suggested to regulate virulence in *Salmonella*, but very little is known about its function or contribution to virulence [107], [108]. The SCC3193 *sirB^−^* mutant was considerably impaired in its capacity to infect axenic tobacco and macerate potato tubers (p<0.001, Figure 5AC), which suggests that *sirB* could play an important role in the virulence of *P. wasabiae* SCC3193. The delayed development of symptoms in tobacco seedlings could be complemented by the addition of the wild-type *sirB* locus (W5S_2384–W5S_2385) in trans (p<0.001). Interestingly, full complementation was also achieved when only the second gene of the locus, *sirB1* (W5S_2384), was used (p<0.001). The addition of the first gene of the locus, *sirB2* (W5S_2385), had no effect when it was added alone. This result suggests that *sirB1*, not *sirB2*, is required for the virulence of SCC3193 and that the phenotype is not due to the polarity of the mutation. We also measured the growth of the *sirB^−^* mutant in planta and found that the mutant showed significantly reduced growth when compared to the wild-type strain (p<0.01, Figure 5B). The addition of the *sirB* locus in trans restored the growth to levels approximating wild-type growth.

In *Salmonella*, the overexpression of the *sirB* locus in trans can suppress effects caused by mutations in *sirA*, which encodes a two-component system response-regulator similar to ExpA (GacA) in *Pectobacterium* [8], [107]. SirA is needed for the transcriptional activation of *Salmonella* invasion genes within the pathogenicity island 1 (SPI1). The *Salmonella* SirB is also necessary for the full expression of SirC (not present in SCC3193), a transcription factor encoded within SPI1 [108]. Although SirC is essential for the invasion of the intestinal epithelium, the *sirB* locus is not needed for the invasive phenotype [108]. Due to its effect on SirC expression and the ability to suppress *sirA* mutant phenotypes, SirB is considered to be a putative transcriptional regulator, although it does not belong to any known class of transcription factors. In *E. coli*, the *sirB* locus is nonessential [109], and no function has been reported. However, the putative operon in which the locus is situated is well characterized and contains genes involved in basic cellular functions, such as protein synthesis and the biosynthesis of LPS [109]–[111]. To our knowledge, SirB has not been reported to regulate the other genes in the operon. An online program for transmembrane helix prediction (TMHMM Server v. 2.0 [112]) predicts the SCC3193 *sirB2* to encode a 14.9-kDa (131 aa) membrane protein with four hydrophobic transmembrane helices; no transmembrane helices are predicted for the 30.7-kDa (269 aa) protein encoded by *sirB1*, indicating that the protein is soluble. Therefore, if the locus encodes for a transcriptional regulator, SirB1 is a strong candidate.

Our results highlight the possibility that SirB is an important regulator of virulence in *P. wasabiae* and possibly in other soft rot bacteria. However, further experimental evidence is needed to confirm that SirB functions as a regulator. In contrast to the reduced maceration levels and reduced growth in planta, the *sirB* mutant appears to exhibit similar in vitro PCWDE production and growth as the wild-type, indicating that SirB could be important for bacterial fitness specifically during infection.

#### Type VI secretion system clusters have overlapping functions in *Pectobacterium wasabiae* SCC3193 during potato infection

The mutagenesis of a type VI secretion system machinery encoding cluster, either T6SS-1 (W5S_0962–W5S_0978) or T6SS-2 (W5S_2418–W5S_2441), did not affect maceration capacity in the potato tuber slice assay (data not shown). However, the T6SS-double mutant showed a reduced level of maceration (p<0.05, Figure 5C), suggesting that the two separate T6SS machineries occupy at least partially overlapping functions in planta. The phenotype of the SCC3193 T6SS-double mutant is similar to that of *P. atrosepticum*, in which mutations of its single T6SS cluster genes decrease its virulence in potato [14]. However, there is a contradictory report regarding the virulence of single-gene T6SS mutants of *P. atrosepticum* [100], which may be partially explained by the different virulence assays used in these studies.

Based on the comparison of the T6SS loci with those in other sequenced bacteria in GenBank using blastn, WGS-tblastn and blastp, it is obvious that the first locus (T6SS-1) is conserved among *Pectobacterium* and many other bacteria. The second locus (T6SS-2) provides the best hit for *P. wasabiae* WPP163, *P. wasabiae* type strain and *Pantoea* and *Erwinia* species; its synteny differs from the first locus. This clearly explains why our island prediction picked T6SS-2, not T6SS-1, as a genomic island of probable horizontal origin (GI_30). T6SS-2 was found to be similar, apart from a few additional ORFs, to a corresponding cluster in *Erwinia amylovora* CFBP1430 (EAMY_3000–EAMY_3028). The same cluster is also present in *E. pyrifoliae* and *E. tasmaniensis* [113]. We were not able to find a typical *Pectobacterium* style T6SS in the available *Erwinia* strains.

Our results support the hypothesis that T6SS contributes to virulence in *Pectobacterium* and that the function of the two T6SS clusters of possibly different evolutionary origins in *P. wasabiae* strains is at least partially redundant. However, we cannot rule out that T6SS in *Pectobacterium* may have another target in addition to plants. The process by which *Pectobacterium* utilizes T6SS during potato colonization and for its potential other targets remains to be elucidated.

#### Virulence cluster 2 contains a putative lipoprotein transport system and a *hopL1*-like gene

**Figure 6:**
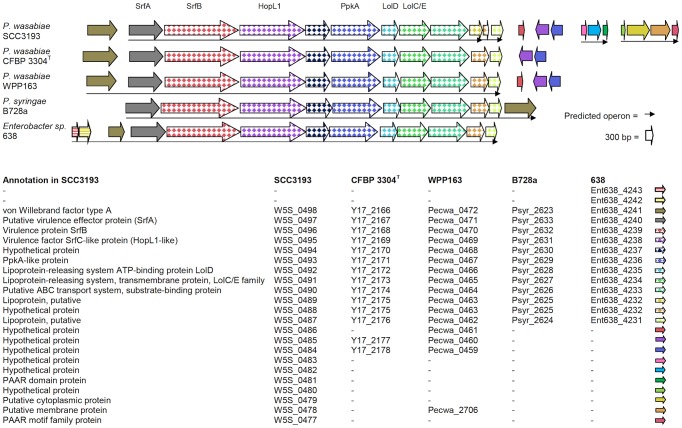
Virulence cluster 2 locus comparison. Visualization and a comparison of the Vic2 locus was completed in *Pectobacterium wasabiae* SCC3193, which contains a putative lipoprotein transporting system and a HopL1-like protein. A comparison of the gene cluster was conducted using blastn and blastp against a nucleotide collection and against non-redundant protein sequences to obtain strains with similar loci. ORFs are indicated using colored and scaled arrows, except in case of *P. wasabiae* CFPB 3304T for which operons were not predicted.

The virulence cluster 2 (Vic2) deletion mutant exhibited a reduced level of maceration (p<0.01, Figure 5C) in the potato tuber slice assay. The respective cluster (W5S_0498–W5S_0477) contains 22 ORFs, has a total size of ∼22.3 kb and has unique features when compared to other bacterial sequences available (Figure 6). The first 12 ORFs (W5S_0498–W5S_0487) form a cluster found in a handful of bacterial species, in addition to *P. wasabiae* strains (SCC3193, WPP163, CFPB 3304^T^), with completed genome sequences, such as *Enterobacter* sp. 638 (Ent638_4231–Ent638_4241), *Xenorhabdus bovienii* (XBJ1_1142–XBJ1_1152), *Azotobacter vinelandii* (Avin_51980–Avin_52100), *Pasteurella multocida* subsp. *multocida* (PM1818–PM1828), *P. syringae* (Psyr_2623–Psyr_2633, PSPTO_2870–PSTO_2880), *Pseudomonas putida* S16 (PPS_0194–PPS_0182) and *Haemophilus parainfluenzae* (PARA_16340–PARA_16450). Notably, this cluster is not found in any soft rot species other than *P. wasabiae*, and it was predicted to be horizontally acquired in SCC3193 (GI_7).

The cluster containing the first 12 ORFs encodes a protein that is similar to HopL1 (query coverage 99%, identity 42–43%, E 0.0), which has been characterized as T3SS secreted in *P. syringae* [114], and a putative lipoprotein transport system (Figure 6). As *P. wasabiae* lacks T3SS, it seems reasonable that HopL1 may be secreted through the putative lipoprotein transport system or some other machinery in SCC3193. We investigated how common it is that T3SS is missing from strains harboring the cluster that carries *hopL1*. Therefore, we compared two core proteins of T3SS derived from *P. syringae* DC3000 (HrpN/HrpU, HrcC) against the genomes of *P. wasabiae* CFPB 3304T, *P. wasabiae* WPP163, *Enterobacter* sp. 638, *X. bovienii*, *A. vinelandii*, *P. multocida* subsp. *multocida*, *P. putida* and *H. parainfluenzae* using tblastn. We were unable to find T3SS-related genes in these strains. The lack of T3SS in *P. wasabiae* WPP163 and other *P. wasabiae* strains tested previously, *Enterobacter* sp. 638, *X. bovienii* and *P. putida* are also noted in the literature [34], [115], [116]. We suggest that HopL1 could be functional without T3SS. In conclusion, we hypothesize that this putative lipoprotein transport system and/or HopL1-like protein may be responsible for the Vic2 deletion mutant phenotype in potato.

#### Virulence cluster 1 contains phage-related genes and a putative type I site-specific restriction-modification system

The Virulence cluster 1 (Vic1) deletion mutant exhibited a reduced capacity to macerate potato tuber slices when compared with the wild-type SCC3193 (p<0.01, Figure 5C), suggesting that one or several of the deleted genes could have an effect on SCC3193 virulence. The ∼14.4-kb genomic region contains ten ORFs (W5S_0467–W5S_0476) and is partially overlapping with GI_6 (W5S_0459–W5S_0472). The region is largely lacking from the genomes of other *Pectobacterium* strains, including *P. wasabiae* WPP163 and CFBP 3304^T^. ORFs W5S_0467 to W5S_0472 are part of a putative phage. The last ORF of Vic1 (W5S_0476) is annotated to encode a toxin component of a toxin-antitoxin system (Fic family) and is classified into COG3943 as a virulence protein, but no specific function for this class of proteins is known.

ORFs W5S_0473–W5S_0475 form a three-gene operon encoding a putative type I restriction modification (R-M) system that is thought to protect bacteria against invading foreign DNA [117]. Restriction-modification systems are common among bacteria, and there are implications of an extensive lateral transfer of the R-M genes [118], [119]. The position of the SCC3193 R-M system between GI_6 and GI_7, and the absence of this system in other *Pectobacterium* strains, including *P. wasabiae* WPP163 and CFBP 3304^T^, implies that the system may have been horizontally acquired in SCC3193. Furthermore, mutation of the type I R-M methyltransferase of *Yersinia pseudotuberculosis* was shown to lead to decreased virulence in a mouse model by an unknown mechanism [120]. Whether the SCC3193 methyltransferase has a role in virulence is a matter of speculation. Further studies are necessary to identify the exact virulence determinant(s) responsible for the observed reduced virulence phenotype of the Vic1 deletion mutant.

### Concluding remarks

The model strain *Pectobacterium* sp. SCC3193 has been one of the most intensively studied soft rot strains for over two decades. Therefore, the molecular-level information on its virulence has had a significant impact on the theory of virulence in *Pectobacterium*. However, SCC3193 was originally incorrectly identified as *P. carotovorum* at the time of isolation, and our extensive phylogenetic analysis in this report reveals that it belongs to the species *P. wasabiae*, which is a less characterized species in the soft rot group. It is likely that other *Pectobacterium* isolates could also be incorrectly classified, and *P. wasabiae* may be more common than previously thought. Our report will facilitate further studies of the distribution and molecular biology of *P. wasabiae*, as a well-studied model strain has been added to the species, and three genome sequences are now publicly available, including the Japanese type strain sequenced in this study.

The absence of the T3SS and T3 effectors is the most distinctive feature separating *Pectobacterium wasabiae* SCC3193 and other *P. wasabiae* strains from other soft rot bacteria, other plant pathogens and other animal pathogenic enterobacteria. However, the number of confirmed T3 effectors is limited in soft rot bacteria overall, compared with the dozens in hemibiotrophs, and the effect of T3SS on virulence is not central in *Pectobacterium*. Our genomic approach, which was supplemented with in planta experiments, revealed putative ways for *Pectobacterium wasabiae* and other pectobacteria to establish infection. Promising candidates for novel virulence determinants having an effect at the early stage of infection are Virulence cluster 2, which carries genes for a putative novel lipoprotein transport system; HopL1, which is an adjacent T3SS effector-like protein; and T6SS. The latter has similar features to T3SS, which injects effectors into the host cell. T6SS is found in a variety of bacterial species and is not confined to pathogens. On the nitrogen-fixing plant symbiont *Rhizobium leguminosarum*, T6SS is related to host specificity [121]. The *P. wasabiae* benzoic acid/salicylic acid methyltransferase may also represent a novel way to manipulate the host in the latent stage, but we have not yet found experimental evidence to support this hypothesis.

In the future, to learn more about the latent stage of *Pectobacterium* infection, we should also consider bacteria outside the pathogens relying on T3SS. Many endophytic bacteria lack T3SS, T4SS and/or the production of pectinolytic enzymes common in phytopathogens [122]–[125]. They are known to use a diverse set of attachment structures and flagella-based motility to colonize plants and to produce plant hormones and other compounds for the modification of plant metabolism. Altogether, endophytic bacteria and symbionts, which efficiently establish interactions with plants, could be viewed as an opportunity to learn more about colonization mechanisms, which may also be important for the pathogenic lifestyle of soft rot bacteria during the latent stage of infection.

## Materials and Methods

### Bacterial strains and growth conditions

In this work, we used *Pectobacterium wasabiae* SCC3193, its derivatives and *Pectobacterium wasabiae* CFBP 3304^T^ for the sequencing and/or biological experiments (Table S3). Standard growth conditions included culturing bacteria on Luria Broth (L3522, Sigma-Aldrich, US) for 1 d at +28°C. The antibiotics chloramphenicol (20 µg/mL) or ampicillin (100 or 150 µg/mL) were added when appropriate.

### Genome sequencing

Genomic DNA of *Pectobacterium wasabiae* CFBP 3304^T^ was extracted from an overnight culture using phenol-ether purification and ethanol precipitation. The quality and quantity of the DNA was assessed using spectrophotometry and agarose gel electrophoresis. The DNA concentration used for the hybridization step was 8 pM. Template amplification/cluster generation was performed using the Cluster station and the Single-Read Cluster generation kit v. 4 (Catalog # GD-103-4001). The sequencing was performed with an Illumina Genome Analyzer IIx using a v. 5 sequencing kit (FC-104-5001). All operations were performed following the manufacturer's protocols (Cluster Station User Guide, Part # 15005236 Rev. B, November 2009, Sequencing Kit v5 Reagent Preparation Guide, Part # 15013595 Rev. A, May 2010). Data generated by Illumina Solexa GAIIx were analyzed with SCS-RTA v. 2.9. Matrix and phasing parameters that were estimated from the PhiX control were used for base-calling. The demultiplexing and conversion of bcl-files to FASTQ-files were performed using OLB v. 1.9.0 and CASAVA GERALD v. 1.7.0. The instrument vendor provided all the software. Adapter sequences were clipped from the reads using cutadapt-tool. Additionally, if an adapter was removed from one read, the other read was shortened to reflect the change when necessary. The read data were assembled with ABySS using the following parameters: −j = 2 k = 60 n = 10 q = 15 ABYSS_OPTIONS = ‘–illumina-quality -c 25 -e 25’.

### Accession number of *Pectobacterium wasabiae* CFBP 3304T

This Whole Genome Shotgun project has been deposited at DDBJ/EMBL/GenBank under the accession AKVS00000000. The version described in this paper is the first version, AKVS01000000.

### ORF annotation

The ORF prediction was completed for CFBP 3304^T^ using the Prodigal gene prediction program [126]. Systematic errors made by Prodigal were corrected, and the intergenic areas were double checked for missed gene predictions with the GenePRIMP program [127] and manual correction. Predicted and corrected protein sequences were then functionally annotated with descriptions (DE), Gene Ontologies (GO) and Enzyme Commission Numbers (EC) using the PANNZER tool (Koskinen et al., in preparation; method is unpublished). The COGnitor tool [128] was used to link sequences into COG database clusters. Finally, functional elements (for example, domains) were searched using the InterProScan tool [129].

### Operon prediction

The annotated ORFs of SCC3193 (Koskinen et al., submitted) were grouped into hypothetical operons using the ofs1.2 software [130]. The grouping was based on link probabilities, which represent the probability of a given ORF to be co-expressed with a downstream gene (see Dataset S2). Based on an *E. coli* benchmark (RegulonDB 6.7 release [131]), our estimates suggest that thresholding these link probabilities at 0.54 will recall approximately 83% of true operon links with 81% specificity. The OFS operon prediction is based on intergenic distances, similarities between annotations and the conservative clustering of similar genes in other species. The set of “other species” (referred to as informant species) used in this analysis was compiled using a single representative of each bacterial order enlisted in the NCBI bacteria taxonomy. At the time of compilation, there were 119 orders with at least one full genome sequenced; therefore, the list contains 119 entries (Dataset S3).

### Phylogenetic tree

In addition to the predicted protein sequences of *Pectobacterium wasabiae* SCC3193 and *Pectobacterium wasabiae* CFBP 3304^T^, full RefSeq proteomes were fetched from NCBI for 52 additional species. One-to-one orthologs of SCC3193 proteins were determined using the RBH (reciprocal best hit) criterion. Protein X.G1 from proteome G1 and protein X.G2 from proteome G2 are reciprocal best hits, if there X.G2 is the best match of X.G1 in proteome G2 and X.G1 is the best match of X.G2 in proteome G1. The full proteomes of SCC3193 and 53 target species were compared using SANS [41] with window size of 100. For phylogenetic analysis, we selected 51 groups of orthologs of SCC3193 proteins present in each of the 53 other species. The ortholog groups were then aligned using Muscle v. 3.8.31 [132]. For all multiple alignments, 1000 bootstrap trees were created using RAxML v. 7.0.4 [133]. The settings used for RAxML were “raxmlHPC -m PROTGAMMAJTT -c 4 -f d -n %s -s %s -x 137 -N 1000”, where %s was replaced with the correct input and corresponding output file names. All the bootstrapped trees were merged using the Consense program v. 3.68 from the Phylip package [134]. The merged trees were visualized using iTOL webtools [135].

### Biochemical tests

Biochemical tests for the ability to reduce sugars, phosphatase activity, indole production, growth on sorbitol, growth on melibiose, growth on raffinose, growth on lactose, utilization of keto-methyl glucoside, growth in 5% NaCl and growth at +37°C were conducted for *P. wasabiae* SCC3193 and the type strains *P. wasabiae* CFBP 3304^T^, *P. carotovorum* CFBP 2046^T^ and *P. atrosepticum* HAMBI 1429^T^ according to the previously described protocols [136], [137].

### Proteome comparison

In the proteome comparison, we re-identified all the ORFs for the sequenced *Pectobacterium* and *Dickeya* species and for the outgroup *Yersinia pestis* CO92. This was performed using Prodigal for gene prediction. The proteomes were then aligned using the blastp program and clustered into orthologous groups with OrthoMCL program. OrthoMCL clusters were converted into an orthologs vs. species (OvsS) matrix. The obtained OvsS matrix can be altered for ease of interpretation by ordering similar columns and rows next to each other, which was accomplished by creating a hierarchical cluster tree from the rows and columns. The internal nodes of the tree were flipped to place more similar neighboring clusters next to each other [138]. The similarity of the columns was based on the Pearson correlation, while the similarity of the rows was based on the cosine similarity. These measures were selected by reviewing the obtained visualization from the different similarity measures. The ordered matrix was then visualized on a heat map. A distance matrix was created by calculating the Pearson correlation similarity scores between species in the OvsS matrix and then visualized as a heat map.

### Multiple genome alignment

*Pectobacterium wasabiae* CFBP 3304^T^ contigs were ordered by aligning them against a reference genome, *Pectobacterium wasabiae* SCC3193. This alignment was created with Mauve v.2.3.1 [139]. The reordered *Pectobacterium wasabiae* CFBP 3304^T^ contigs were then aligned against the genomes of *Pectobacterium wasabiae* WPP163 and *Pectobacterium atrosepticum* SCRI1043. Mauve produced a genome content distance matrix as an output from the pairwise alignments, which was used to quantify the differences in the aligned sequences.

### Genomic islands

Genomic island predictions were performed using three computational tools: two that utilize sequence composition-based GI prediction methods, SIGI-HMM from the Colombo package [63] and IslandPath-DIMOB [64], and one that is based on a comparative genomic-based GI prediction, IslandPick [65]. Automated predictions were manually curated. The borders were adjusted such that genes known to be frequently associated with GIs or mobile genetic elements, such as integrase and phage genes, were added when these genes were located adjacent to automatically predicted islands. In addition, when necessary, the proximity of tRNA genes, similar functions encoded adjacent to the predicted island and the absence of the region in closely related strains based on blastn were used as criteria to modify the islands. Finally, we filtered out all islands consisting of less than five ORFs. To determine whether the SCC3193 genomic islands are also present in the genomes of other soft rot bacteria, blastn searches were performed with the nucleotide sequences of the islands against all available *Pectobacterium* and *Dickeya* genomes in GenBank. For *P. carotovorum* WPP14 and *P. carotovorum* subsp. *brasiliensis* PBR1692, whose genomes have not been completed, the combined query coverage of all contigs for each island was estimated based on a WGS-blastn search against these genomes. For *P. wasabiae* CFBP 3304^T^, the estimation was based on blastn pairwise alignment.

### Genome comparisons

The comparison of orthologous proteins of *Pectobacterium wasabiae* SCC3193 was conducted against 41 selected soft rot bacteria, plant pathogens, animal pathogens and insect pathogens (Dataset S4) to identify novel virulence genes in SCC3193, in *P. wasabiae* or in soft rot bacteria. The comparison was performed manually, based on clusters created with OrthoMCL [140]. The genome and specific proteins identified were analyzed using the genome viewer Argo v. 1.0.31 [141], operon predictions, tblastn via Embster 2.0 beta launched from the CSC Chipster platform (http://chipster.csc.fi/embster/), annotation, blastn and blastp in GenBank NCBI [142], [143]. These analyses were conducted to identify known virulence determinants, detect missing virulence determinants and identify novel putative virulence determinants. The analysis of PCWDEs was performed by utilizing publications, sequence similarity and selected GO terms (Table S4) known to be associated with the enzymes of interest. The enzymes with the selected GO terms were mined using PANNZER (Koskinen et al., in preparation; method is unpublished), InterProScan [129] or BioMart [144]. The proteinases present in *E. coli* were discarded according to Glasner et al. [22], and they were not considered as putative enzymes targeting plants.

### Targeted mutagenesis

The inactivation of individual genes and gene clusters was performed by deleting target sequences and replacing them with an antibiotic cassette, according to Datsenko and Wanner [145]. The antibiotic cassette was amplified from a pKD3 template plasmid with primers carrying 50 bp of similar sequence to the genomic DNA of *Pectobacterium wasabiae* SCC3193 (Table S5). The cloning was conducted using the proofreading PCR enzyme Phusion according to the manufacturer's 3-step protocol (Finnzymes). The insert was gel purified. For the electrocompetent cells, bacteria were grown overnight, diluted 1∶50 and grown to OD_600_ 0.4. The cells were cooled down and washed twice with sterile ice-cold ddH_2_O and once with sterile ice-cold 10% glycerol. The cells were resuspended in 1.5–2x volume of sterile ice-cold 10% glycerol. Electroporation was performed with the following settings: 2.5 kV, 25 µF and 200 Ω in 0.2 cm cuvettes (Bio-Rad Laboratories). The recovery times for pKD46 and the antibiotic cassette insertion were 15 min and 3.5 h, respectively. The mutations and the position of the insert were confirmed with two PCR reactions according to Datsenko and Wanner [145] in addition to sequencing (Table S5). For the double mutant of T6SS, the first antibiotic cassette was digested using flipase produced in trans from the pFLP2 plasmid [146].

### Plasmid constructs

For the complementation experiments, the genes of the *sirB* locus were amplified by PCR from wild-type SCC3193 genomic DNA using the proofreading PCR enzyme Phusion (Finnzymes). The following primers were utilized: SirB1_compl_F and SirB1_compl_R for *sirB1*; SirB2_compl_F and SirB2_compl_R for *sirB2*; and SirB2_compl_F and SirB1_compl_R for the complete *sirB* locus (Table S5). The PCR products were gel purified, digested with HindIII and SacI and ligated into pMW119 (Nippon Gene), which was digested with the corresponding enzymes. The constructs were confirmed via PCR and sequencing.

### Tobacco seedling virulence assay

The bacterial virulence was tested on axenic tobacco seedlings (*Nicotiana tabacum* cv. ‘Samsun’) according to Pirhonen et al. [24]. The seedlings were propagated in 24-well tissue culture plates on ½ MS medium supplemented with vitamins (Duchefa) and 2% sucrose and solidified with 0.8% agar. The seedlings were grown for 16 days with a 16/8 h day/night cycle at 26/22°C. The bacterial strains were grown overnight, washed and diluted in 10 mM MgSO_4_. A single leaf from each of 48 plants was wounded with a needle and inoculated with 1.5 µl of bacterial solution (OD_600_ 0.05). The inoculated plants were kept in the dark at room temperature, and the development of disease symptoms (leaf maceration) was scored visually at 24 and 48 h after inoculation on a scale of 0 to 6 according to the severity of symptoms. To determine bacterial growth in planta (cfu/plant), the inoculated plants were kept in the dark at 26°C and homogenized into 10 mM MgSO_4_ after 24 and 44 h; serial dilutions of bacteria were plated. All experiments were repeated three times, and the data of each replicate were analyzed statistically utilizing a Mann-Whitney significance test for the pairwise comparison of two independent samples using PASW Statistics 18.

### Potato tuber slice virulence assay

For the tuber slice assay [17], bacterial strains were grown overnight, washed and resuspended into 10 mM MgSO_4_. Tubers (cv. Van Gogh, H&H Tuominen, Finland) were washed with tap water and surface sterilized with 10% Na-hypochlorite for 5 min. The tubers were sliced 0.6 cm thick, surface sterilized again with flaming and placed on wet paper tissue in a Petri dish. Ten to 12 tuber slices were inoculated with 10 µl of bacterial solution containing 10^6^ cfu/ml and incubated at room temperature in a shady place for three days. The macerated area was assessed, and the results were classified into seven groups: 0%, ∼5%, ∼20%, ∼50%, ∼75%, ∼90% and 100% rotten tissue per tuber slice surface area. The experiments were repeated five times, and data from all the replicates were combined for statistical analyses utilizing the Mann-Whitney significance test for the pairwise comparison of two independent samples using PASW Statistics 18.

### Enzyme assays and growth curve

Cellulase (Cel) and polygalacturonase (PehA) activities were assayed from 10 µl of supernatant of liquid cultures grown overnight and from appropriate dilutions of the supernatant on corresponding enzyme indicator plates [7]. The growth of the *sirB*, Vic1, Vic2 and T6SS-double mutants were compared with the SCC3193 wild-type strain in an *hrp*-inducing minimal medium [147] using 0.4% polygalacturonic acid (Sigma P3850) as a sole carbon source. The bacteria were washed once with 1x minimal medium, diluted to OD_600_ 0.1 and then grown for 20 h at +28°C with shaking (200 rpm).

## Supporting Information

Dataset S1 Protein clusters used for construction of the phylogenetic tree.
(XLS)

Dataset S2 Operon prediction.
(XLS)

Dataset S3 Informant species used in operon prediction.
(XLS)

Dataset S4 Bacterial strains used in SCC3193 ortholog comparison.
(DOC)

Table S1 Plant cell wall-degrading enzyme comparison.
(DOC)

Table S2 Genomic islands in *Pectobacterium wasabiae* SCC3193.
(XLS)

Table S3 Strains used in biological experiments.
(DOC)

Table S4 GO terms for plant cell wall-degrading enzymes.
(DOC)

Table S5 Primers for cloning and sequencing.
(DOC)
