# Supplementary material for: Revised Phylogeny and Novel Horizontally Acquired Virulence Determinants of the Model Soft Rot Phytopathogen Pectobacterium wasabiae SCC3193
Source: PLoS Pathog. 2012 Nov 1;8(11):e1003013. doi: 10.1371/journal.ppat.1003013 (PMC3486870; doi:10.1371/journal.ppat.1003013)
Supplement: Dataset S4 — Bacterial strains used in SCC3193 ortholog comparison. (DOC) [file ppat.1003013.s004.doc]

*Aeromonas hydrophila* subsp. *hydrophila* ATCC 7966

*Aeromonas salmonicida* subsp. *salmonicida* A449

*Dickeya dadantii* 3937

*Dickeya dadantii* Ech586

*Dickeya dadantii* Ech703

*Dickeya zeae* Ech1591

*Erwinia amylovora* ATCC 49946

*Erwinia pyrifoliae* Ep1/96

*Erwinia tasmaniensis* Et1/99

*Escherichia coli* B str. REL606

*Escherichia coli* O157:H7 str. EDL933

*Legionella pneumophila* str. Lens

*Legionella pneumophila* str. Paris

*Legionella pneumophila* subsp. *pneumophila* str. Philadelphia 1

*Pantoea* sp. At-9b

*Pantoea stewartii* subsp. *stewartii* DC283

*Pectobacterium atrosepticum* SCRI1043

*Pectobacterium carotovorum* subsp. *brasiliensis* PBR1692

*Pectobacterium carotovorum* subsp. *carotovorum* PC1

*Pectobacterium carotovorum* subsp. *carotovorum* WPP14

*Pectobacterium wasabiae* WPP163

*Photorhabdus luminescens* subsp. *laumondii* TTO1

*Pseudomonas syringae* pv. *phaseolicola* str. 1448A

*Pseudomonas syringae* pv. *syringae* str. B728a

*Pseudomonas syringae* pv. *tomato* str. DC3000

*Ralstonia solanacearum* GMI1000

*Ralstonia solanacearum* UW551

*Salmonella enterica* subsp. *enterica* serovar Paratyphi A str. AKU_12601

*Salmonella enterica* subsp. *enterica* serovar Typhi str. CT18

*Salmonella enterica* subsp. *enterica* serovar Typhimurium str. LT2

*Shigella flexneri* 2a str. 2457T

*Xanthomonas campestris* pv. *campestris* str. B100

*Xanthomonas campestris* pv. *campestris* str. 8004

*Xanthomonas campestris* pv. *campestris* str. ATCC 33913

*Xanthomonas campestris* pv. *vesicatoria* str. 85-10

*Xanthomonas oryzae* pv. *oryzae* KACC10331

*Xenorhabdus nematophila* ATCC 19061

*Xylella fastidiosa* Temecula1

*Yersinia pestis* CO92

*Yersinia pestis* Nepal516

*Yersinia pseudotuberculosis* YPIII
