# Supplementary material for: Revised Phylogeny and Novel Horizontally Acquired Virulence Determinants of the Model Soft Rot Phytopathogen Pectobacterium wasabiae SCC3193
Source: PLoS Pathog. 2012 Nov 1;8(11):e1003013. doi: 10.1371/journal.ppat.1003013 (PMC3486870; doi:10.1371/journal.ppat.1003013)
Supplement: Table S1 — Plant cell wall-degrading enzyme comparison. (DOC) [file ppat.1003013.s005.doc]

**Table S1. Plant cell wall-degrading enzyme comparison.** *Pectobacterium* strains produce pectinases, cellulases and proteinases necessary for pathogenicity. The strains share similar enzyme collections with little variation. The enzymes were chosen based on the published literature and GO terms using Pannzer, InterProScan and BioMart. The putative novel proteinases were first selected based on GO terms and the published literature. Proteinases with a homolog in *E. coli* strains according to Glasner et al. (2008) were then extracted. N/D = not detected.

| **Enzyme class** | **SCC3193** | **WPP163** | **CFBP 3304^T^** | **SCRI1043** | **WPP14** | **PC1** | **PBR1692** |
| --- | --- | --- | --- | --- | --- | --- | --- |
| **Pectate lyases** |  |  |  |  |  |  |  |
| PelL | W5S_1993 | Pecwa_2045 | Y17_0324 | ECA2553 | GI:227329374 | PC1_1773 | GI:227111950 |
| PelW | W5S_2145 | Pecwa_2201 | Y17_0918 | ECA2402 | ADT-0002894/5 | PC1_1908 | GI:227111525 |
|  | W5S_2486 | Pecwa_2511 | Y17_3510 | ECA2135 | GI:227326249 | PC1_2170 | GI:227115082 |
| PelI | W5S_3322 | Pecwa_3322 | Y17_4295 | ECA1094 | GI:227327232 | PC1_0988 | GI:227113342 |
| PelA | W5S_4197 | Pecwa_4035 | Y17_1229 | ECA4067 | GI:227326796 | PC1_3858 | GI:227112125 |
| PelB | W5S_4198 | Pecwa_4036 | Y17_1228 | ECA4068 | GI:227326797 | PC1_3859 | GI:227112126 |
| PelC | W5S_4199 | Pecwa_4037 | Y17_1227 | ECA4069 | GI:227326798 | PC1_3860 | GI:227112127 |
| PelZ | W5S_4200 | Pecwa_4038 | Y17_1226 | ECA4070 | GI:227326799 | PC1_3861 | GI:227112128 |
| PelX | W5S_4756 | Pecwa_4562 | Y17_4589 | ECA4510 | GI:227327854 | PC1_4259 | GI:227113921 |
|  | N/D | N/D | N/D | ECA3112 | GI:227327886 | PC1_2863 | GI:227114136 |
| HrpW | N/D | N/D | N/D | ECA2112 | GI:227329951 | PC1_2182 | GI:227114196 |
| **Pectin lyases** |  |  |  |  |  |  |  |
| Pnl | W5S_1693 | Pecwa_1777 | Y17_2017 | ECA1499 | GI:227327190 | PC1_1371 | GI:227115198 |
| **Pectin methylesterases** |  |  |  |  |  |  |  |
| PemA | W5S_1140 | Pecwa_1228 | Y17_1644 | ECA3253 | ADT-0003065 | PC1_3047 | GI:227112721 |
| PemB | W5S_4612 | Pecwa_4427 | Y17_0498 | ECA0107 | GI:227328020 | PC1_4146 | GI:227114480 |
| **Pectin acetylesterase** |  |  |  |  |  |  |  |
| PaeX | W5S_2138 | Pecwa_2195 | Y17_0924 | ECA2408 | GI:227327031 | PC1_1902 | GI:227111531 |
| **Polygalacturonases** |  |  |  |  |  |  |  |
| PehX | W5S_1295 | Pecwa_1410 | Y17_1495 | ECA3111 | GI:227327885 | PC1_2862 | GI:227114135 |
| PehN | W5S_3224 | Pecwa_3223 | Y17_4393 | ECA1190 | GI:227329691 | PC1_1092 | GI:227114428 |
| PehA | W5S_3321 | Pecwa_3321 | Y17_4296 | ECA1095 | GI:227327233 | PC1_0989 | GI:227113343 |
| PehK | N/D | N/D | N/D | ECA3552 | GI:227329732 | PC1_3371 | GI:227114087 |
|  | N/D | N/D | N/D | N/D | N/D | PC1_2125 | N/D |
| **Oligogalacturonide lyase** |  |  |  |  |  |  |  |
| Ogl | W5S_2119 | Pecwa_2176 | Y17_0943 | ECA2426 | GI:227327049 | PC1_1884 | GI:227111549 |
| **Rhamnogalacturonate lyase** |  |  |  |  |  |  |  |
| RhiE | W5S_0803 | Pecwa_0910 | Y17_2411 | ECA0804 | GI:227326316 | PC1_0682 | GI:227113492 |
| **Cellulases** |  |  |  |  |  |  |  |
| BcsZ | W5S_0072 | Pecwa_0071 | Y17_4504  Y17_4720 | ECA4373 | GI:227328208 | PC1_0074 | GI:227113871 |
| CelV | W5S_2584 | Pecwa_2612 | Y17_2707 | ECA1981 | GI:227326352 | PC1_2337 | GI:227111850 |
| CelS | W5S_2951 | Pecwa_2966 | Y17_2916 | ECA2827 | GI:227328809 | PC1_1503 | GI:227112992 |
| **Proteases** |  |  |  |  |  |  |  |
|  | W5S_0443 | Pecwa_0429 | Y17_3267 | ECA0388 | GI:227327163 | PC1_0378 | GI:227114613 |
|  | W5S_0588 | Pecwa_0567 | Y17_2093 | N/D | N/D | PC1_0472 | GI:227115474  GI:227115327 |
|  | W5S_0802 | Pecwa_0909 | Y17_2410 | ECA0803 | GI:227326315 | PC1_0681 | GI:227113491 |
|  | W5S_0893 | Pecwa_0998 | Y17_2472 | ECA0879 | GI:227328244 | PC1_0761 | GI:227114530 |
| Prt1 | W5S_1178 | Pecwa_1266 | Y17_1605 | ECA3211 | GI:227328666 | PC1_3006 | GI:227112677 |
|  | W5S_1276 | N/D | Y17_1512 | N/D | N/D | N/D | N/D |
|  | W5S_2038 | Pecwa_2090 | N/D | ECA2515 | N/D | N/D | N/D |
|  | W5S_2505 | Pecwa_2529 | Y17_2617 | ECA2074 | GI:227328485 | PC1_2217 | GI:227114163 |
|  | W5S_2577 | Pecwa_2605 | Y17_2700 | ECA1988 | GI:227326364 | PC1_2325 | GI:227111862 |
|  | W5S_2866 | Pecwa_2881 | Y17_2835 | ECA2771 | GI:227325926 | PC1_1581 | GI:227115309 |
| PrtW | W5S_2896 | Pecwa_2909 | Y17_2862 | ECA2785 | GI:227326952 | PC1_1553 | GI:227113041 |
|  | W5S_2918 | Pecwa_2931 | Y17_2886 | ECA2802 | GI:227326970 | PC1_1530 | GI:227113022 |
|  | W5S_2995 | Pecwa_3008 | Y17_3469 | ECA1450 | GI:227326714 | PC1_1326 | GI:227113165 |
|  | W5S_3164 | Pecwa_3163 | Y17_4456 | ECA1290 | GI:227329726 | PC1_1168 | GI:227114401 |
|  | W5S_3893 | Pecwa_3747 | Y17_3895 | ECA3785 | GI:227329232 | PC1_3562 | GI:227113953 |
|  | W5S_4080 | Pecwa_3929 | Y17_2986 | N/D | N/D | N/D | N/D |
|  | W5S_4412 | Pecwa_4236 | Y17_3170 | N/D | N/D | N/D | N/D |
|  | N/D | N/D | Y17_2555 | N/D | N/D | N/D | N/D |
|  | N/D | N/D | N/D | ECA0980 | N/D | N/D | N/D |
|  | N/D | N/D | N/D | ECA2007 | GI:227326382 | PC1_2287 | GI:227111881 |
|  | N/D | N/D | N/D | ECA2163 | GI:227326279 | PC1_2143 | N/D |
|  | N/D | N/D | Y17_1313 | N/D | GI:227327451 | PC1_2675 | GI:227112230 |
|  | N/D | N/D | N/D | N/D | GI:227328283 | N/D | N/D |
|  | N/D | N/D | N/D | N/D | GI:227328318 | N/D | N/D |
|  | N/D | N/D | N/D | N/D | GI:227328319 | PC1_1156 | GI:227114389  GI:227112588 |
|  | N/D | N/D | N/D | N/D | N/D | PC1_1154 | N/D |
|  | N/D | N/D | N/D | N/D | N/D | N/D | GI:227112252 |
| **Enzyme class** | **SCC3193** | **WPP163** | **CFBP 3304^T^** | **SCRI1043** | **WPP14** | **PC1** | **PBR1692** |
