# Supplementary material for: Revised Phylogeny and Novel Horizontally Acquired Virulence Determinants of the Model Soft Rot Phytopathogen Pectobacterium wasabiae SCC3193
Source: PLoS Pathog. 2012 Nov 1;8(11):e1003013. doi: 10.1371/journal.ppat.1003013 (PMC3486870; doi:10.1371/journal.ppat.1003013)
Supplement: Table S3 — Strains used in biological experiments. (DOC) [file ppat.1003013.s007.doc]

**Table S3. Strains used in biological experiments.**

| Strain | Genotype | Reference |
| --- | --- | --- |
| *Pectobacterium wasabiae* SCC3193 | wild type | Pirhonen et al 1988 |
| *Pectobacterium wasabiae* T6SS-1 | ΔT6SS-1::cm in SCC3193 background, Cm^R^ | This work |
| *Pectobacterium wasabiae* T6SS-2 | ΔT6SS-2::cm in SCC3193 background, Cm^R^ | This work |
| *Pectobacterium wasabiae* T6SS-d | ΔT6SS-2::cm in ΔT6SS-1 background, Cm^R^ | This work |
| *Pectobacterium wasabiae* SirB | Δ*sirB*::cm in SCC3193 background, Cm^R^ | This work |
| *Pectobacterium wasabiae* Vic1 | ΔVic1::cm in SCC3193 background, Cm^R^ | This work |
| *Pectobacterium wasabiae* Vic2 | ΔVic2::cm in SCC3193 background, Cm^R^ | This work |
