# Supplementary material for: Revised Phylogeny and Novel Horizontally Acquired Virulence Determinants of the Model Soft Rot Phytopathogen Pectobacterium wasabiae SCC3193
Source: PLoS Pathog. 2012 Nov 1;8(11):e1003013. doi: 10.1371/journal.ppat.1003013 (PMC3486870; doi:10.1371/journal.ppat.1003013)
Supplement: Table S4 — GO terms for plant cell wall-degrading enzymes. (DOC) [file ppat.1003013.s008.doc]

**Table S4. GO terms for plant cell wall-degrading enzymes.** GO terms and their descriptions and corresponding EC numbers (if available). These were used to compare the known and predicted plant cell wall-degrading enzymes of the *Pectobacterium* species.

| GO term | GO description | EC number |
| --- | --- | --- |
| GO:0030570 | pectate lyase activity | EC 4.2.2.2 |
| GO:0047489 | pectate disaccharide-lyase activity | EC 4.2.2.9 |
| GO:0047490 | pectin lyase activity | EC 4.2.2.10 |
| GO:0030599 | pectinesterase activity | EC 3.1.1.11 |
| GO:0047911 | galacturan 1,4-alpha-galacturonidase activity | EC 3.2.1.67 |
| GO:0004650 | polygalacturonase activity | EC 3.2.1.15 |
| GO:0047487 | oligogalacturonide lyase activity | EC 4.2.2.6 |
| GO:0000272 | polysaccharide catabolic process | EC 4.2.2.6 |
| GO:0045490 | pectin catabolic process | - |
| GO:0008810 | cellulase activity | EC 3.2.1.4 |
| GO:0016162 | cellulose 1,4-beta-cellobiosidase activity | EC 3.2.1.91 |
| GO:0031217 | glucan 1,4-beta-glucosidase activity | EC 3.2.1.74 |
| GO:0006508 | proteolysis | - |
| GO:0008233 | peptidase activity | - |
| GO:0008237 | metallopeptidase activity | - |
| GO:0004222 | metalloendopeptidase activity | - |
| GO:0004175 | endopeptidase activity | - |
| GO:0008238 | exopeptidase activity | - |
