# Supplementary material for: Revised Phylogeny and Novel Horizontally Acquired Virulence Determinants of the Model Soft Rot Phytopathogen Pectobacterium wasabiae SCC3193
Source: PLoS Pathog. 2012 Nov 1;8(11):e1003013. doi: 10.1371/journal.ppat.1003013 (PMC3486870; doi:10.1371/journal.ppat.1003013)
Supplement: Table S5 — Primers for cloning and sequencing. (DOC) [file ppat.1003013.s009.doc]

**Table S5. Primers for mutagenization, cloning and sequencing.**

| **Primer** | **Sequence** | **Reference** |
| --- | --- | --- |
| **Mutagenization primers** | | |
| ΔT6SS-1F | AATCGACTTTACTTCTCCGGGCCCGAGTGCCCTTACGTTGGTATAGACACGTGTAGGCTGGAGCTGCTTC | This work |
| ΔT6SS-1R | ATCATCTTGGTGAATGTCCGGTTCCGATATTAATAATTTACTATCAGGGACATATGAATATCCTCCTTAG | This work |
| ΔT6SS-2F | CTGTGACTCGCCTTGTTCATAACCGGCCTTCGGCCATTTTTTAGGCATTAGTGTAGGCTGGAGCTGCTTC | This work |
| ΔT6SS-2R | GTAAAAAATAGCCGGAAGGATCGTTGGGATCGCTTCCGGCTATTATTAAACATATGAATATCCTCCTTAG | This work |
| ΔSirBF | ATAAATTAACGCTGCATTACTGCAAAACGGTTTCTTAATGTAAGACTGTTGTGTAGGCTGGAGCTGCTTC | This work |
| ΔSirBR | CCGGGTGTCGTTAGGGCGCTGGTTGAATACCATCAATTATTAGGAGCAATATGGGAATTAGCCATGGTCC |  |
| ΔVic1F | TGAAAAGCGAGTAATTTAGCACCAAGTGCTATAGTTAAGGTTACAGGATGGTGTAGGCTGGAGCTGCTTC | This work |
| ΔVic1R | GCTCGGCTGGTGAGTCATGTCAGTGCAGGAATCTCAGTGCTGTATTCTTCCATATGAATATCCTCCTTAG | This work |
| ΔVic2F | GTAGTATCATCCGCTCTGTTTAGGGTGGAGTTATTACATTGCCATCACTAGTGTAGGCTGGAGCTGCTTC | This work |
| ΔVic2R | ATTGGCTTTCATTCTCAATCATATCAATCTACGTCGATAGCGTTGCTGCCCATATGAATATCCTCCTTAG | This work |
| **Confirmation primers** | | |
| C2 | GATCTTCCGTCACAGGTAGG | Datsenko and Wanner 2000 |
| C1 | TTATACGCAAGGCGACAAGG | Datsenko and Wanner 2000 |
| T6SS1F | TTGGTGCGAGTCTTTCACACCGAT | This work |
| T6SS1R | TCACCACATCACGGATTATCGCCA | This work |
| T6SS2F | AGGCTGTTTAGGATCACTCGCCAA | This work |
| T6SS2R | CCGTGTTTGGCACTCAGCTTTGAT | This work |
| SirBF | GTTGGATTTAGGCACTGGAA | This work |
| SirBR | CGGTTAGCCTTATCGAATGA | This work |
| Vic1F | TTACCGCGCTTCTTAATCAC | This work |
| Vic1R | GGCTGTAGCGTGTCATCATC | This work |
| Vic2F | GAGAAGAGAAACGAGCCTGA | This work |
| Vic2R | CTGAGATTCCTGCACTGACA | This work |
| **Complementation primers** | | |
| SirB1_compl_F | ACAAGCTTGAAAAACTGTTTGGCGTTATC | This work |
| SirB2_compl_F | CCAAGCTTCGCGAGGATTTACACAACTA | This work |
| SirB1_compl_R | ACGAGCTCCCAAACAGTACAAAAGGCAGA | This work |
| SirB2_compl_R | ACGAGCTCCACTCATAATTATTCCATCAGCA | This work |
| **Sequencing primers for complementation constructs** | | |
| pMW119(seq)_Fw | CTCACTCATTAGGCACCCCA | This work |
| pMW119(seq)_Rv | GCCTCTTCGCTATTACGCCA | This work |
